# In Vivo Therapeutic Potential of Biologically Synthesized Nanoparticles From Pine Needle Leaf Extract in Streptozotocin‐Induced Diabetic Rats

**DOI:** 10.1155/bmri/1938383

**Published:** 2026-01-21

**Authors:** Nourhane A. Darwich, Noura S. Abouzeinab, Ahmed F. El-Sayed, Rana El Hajj, Mahmoud I. Khalil

**Affiliations:** ^1^ Department of Biological Sciences, Faculty of Science, Beirut Arab University, Beirut, Lebanon, bau.edu.lb; ^2^ Microbial Genetics Department, Biotechnology Research Institute, National Research Centre, Giza, Egypt, nrc.sci.eg; ^3^ Egypt Center for Research and Regenerative Medicine (ECRRM), Cairo, Egypt; ^4^ Molecular Biology Unit, Department of Zoology, Faculty of Science, Alexandria University, Alexandria, Egypt, alexu.edu.eg

**Keywords:** antihyperglycemic, anti-hyperlipidemic, diabetes mellitus, green-synthesized nanoparticles, in silico docking, streptozotocin (STZ)

## Abstract

**Background:**

Diabetes mellitus (DM) is one of the most widespread metabolic diseases characterized by increased blood glucose levels. According to the most recent research, the treatment of diabetes could be improved with the use of green‐synthesized nanoparticles due to their biocompatibility, efficient cellular uptake, and targeted therapy.

**Objective:**

In the present study, nanoparticles were biosynthesized using pine needle leaf extract to assess their antidiabetic potential in a streptozotocin (STZ)‐induced diabetic rat model.

**Methods:**

Initially, silver nanoparticles (AgNPs), yttrium‐doped AgNPs (Y‐AgNPs), and gadolinium–chromium–yttrium–doped AgNPs (GCY‐AgNPs) were green‐synthesized using pine needle leaf extract and characterized by UV‐Vis, PL, XRD, FTIR, SEM‐EDX, TEM, and VSM. Diabetes was induced in male Sprague–Dawley rats by a single intraperitoneal injection of STZ (55 mg/kg), followed by administration of pine needle leaf extract (PNLE), AgNPs, Y‐AgNPs, GCY‐AgNPs (7.5 mg/kg each), and glibenclamide (GLB, 5 mg/kg) to diabetic rats for 7 or 21 days. The assessment included body weight, blood glucose levels, and biochemical, lipid, and kidney histology, along with molecular docking for nanoparticle–protein interactions.

**Results:**

Diabetic rats exhibited weight reduction alongside increased blood glucose levels. Treatment with green‐synthesized nanoparticles markedly reduced blood glucose, along with aminotransferase (AST), alanine aminotransferase (ALT), urea, creatinine, serum triglycerides (TG), total serum cholesterol (TC), very low‐density lipoprotein (VLDL), and low‐density lipoprotein (LDL), while increasing high‐density lipoprotein (HDL) levels. The intraperitoneal injection of green‐synthesized nanoparticles provided notable protection to rat kidneys against STZ‐induced damage by maintaining the cortical and tubular structures, as well as mitigating the histopathological lesions. In‐silico docking studies confirmed strong interactions of the nanoparticles with the antidiabetic targets via hydrogen and hydrophobic interactions, revealing possible therapeutic applications.

**Conclusion:**

Among all nanoparticle formulations, GCY‐AgNPS showed the strongest protective effect against STZ‐induced diabetic kidney damage. The findings exhibited significant antidiabetic and antihyperlipidemic effects against STZ‐induced diabetes in rats.

## 1. Introduction

Diabetes is a chronic, life‐threatening metabolic disorder characterized by high blood glucose levels. The elevated glucose levels arise from inadequate synthesis or insulin release from the pancreas, excessive production of glucagon, or low responsiveness to insulin in peripheral tissues. These disturbances primarily affect lipid, carbohydrate, and protein metabolism [1, 2] The International Diabetes Federation (IDF) reports a growing trend in diabetes cases, with 537 million individuals worldwide. This is predicted to reach 643 million in 2030 and 783 million in 2045 [3, 4].

Diabetes mellitus can be classified into three main types: type 1, type 2, and gestational diabetes mellitus (GDM), with the majority of cases being type 2 diabetes. In type 1 diabetes, the immune system attacks and damages the pancreatic beta cells responsible for insulin production. Type 2 diabetes (T2D) is a primary global health concern. Primarily caused by insufficient insulin production by pancreatic *β*‐cells, coupled with insulin resistance in the liver, muscle, and adipose tissue [5]. However, gestational diabetes occurs during pregnancy when blood sugar levels become elevated [6].

Although diabetes mellitus can be asymptomatic, it remains a serious health condition that can lead to severe complications, including nephropathy, cardiomyopathy, coronary artery disease, retinopathy, and stroke [7]. While various risk factors led to the development of diabetes, maintaining glucose control remains at the core of the therapeutic management of the condition. Nevertheless, the development of new therapeutic approaches is crucial for improving the efficiency of regulating diabetes [7].

Currently, commercially available antidiabetic drugs include biguanides, sulphonylureas, alpha‐glucosidase inhibitors, meglitinides, and thiazolidinediones. However, these medications have been linked with a variety of side effects, such as hypoglycemia, diarrhea, nausea, fluid retention, and bloating [8]. This cause has prompted the development of new antidiabetic drugs with fewer adverse reactions. Despite the lack of established safety and efficacy for these commercially available drugs, humans have used several herbs to manage diabetes [9]. Plant‐based therapeutics are generally considered safer, with minimal toxicity or adverse effects [10]. Innovative technologies, such as nanoparticulate systems, are currently being investigated to improve the effectiveness of antidiabetic treatments [11, 12]. Among these, nanoparticles (NPs) derived from plant extracts possess notable potential for enhancing drug bioavailability [13]. Pine trees are monoecious woody plants with needle‐shaped evergreen leaves that thrive in diverse environments, making them a potential source for innovative plant‐based treatments [14].

NPs and nanomaterials have gained significant attention in various fields, such as the biomedical and food technology sectors, prompting researchers’ attention to nanoscience [14]. Silver NPs (AgNPs) possess unique physicochemical properties, making them more significant in biological applications, considering their biosafety. AgNPs are widely utilized as anticancer, antibacterial, and biosensors for managing diabetes mellitus [15]. The green synthesis technique of metallic NPs offers distinct advantages over traditional, conventional chemical and physical methods, including cost‐effectiveness, simplicity, and environmental sustainability [16, 17].

Recent investigative work has indicated that certain metal NPs may possess intrinsic antidiabetic properties that are independent of their use as drug carriers. Reports show that green‐synthesized silver NPs significantly lower blood glucose, enhance antioxidant defenses, and shield STZ‐induced diabetic model pancreatic *β*‐cells from damage due to oxidative stress and aberrant insulin signaling [18]. Additionally, the rare‐earth elements yttrium and gadolinium, which have been shown to possess promising biological properties and catalytic activity, may help enhance glucose control and provide some degree of tissue protection in certain metabolic disorders [19]. These findings underscore the possible therapeutic applications of metallic and rare‐earth–based NPs as direct antidiabetic agents.

In line with our goal to improve the activity and sensitivity of naturally synthesized NPs, new approaches using plant extracts are presently being investigated [16, 17]. This study aimed to investigate the antidiabetic potential of NPs synthesized from pine needle leaf extract in a streptozotocin (STZ)‐induced diabetic rat model. Specifically, it examined their effects on liver and kidney biomarkers, lipid profile, fasting blood sugar (FBS), and histological impact on the kidney. Additionally, molecular docking examination was performed to study the binding interactions between the NPs and antidiabetic molecular targets, thereby providing an innovative approach to diabetes treatment.

## 2. Materials and Methods

### 2.1. Green Synthesis and Characterization of NPs

For the biosynthesis of the NPs, a 1‐mM aqueous solution of the metallic precursor compounds—silver nitrate (AgNO₃), yttrium nitrate hexahydrate (Y(NO₃)₃·6H₂O), and gadolinium nitrate hexahydrate (Gd(NO₃)₃·6H₂O)—was prepared. The pine needle leaf extract (PNLE) was obtained by boiling 6 g of finely chopped pine needles in 250 mL of distilled water for 20 min, after which it was cooled, filtered, and the volume was made up to 300 mL with distilled water. For synthesis, 50 mL of the precursor solution was added to 250 mL of pine needle leaf extract, which was then heated to 60°C for 30 min, followed by a 90‐minute dark incubation period, during which a color transition signaled NP development. A color change signifying the creation of NPs was noted, followed by centrifugation of the product and washing at 10,000 rpm for 30 min. The product was then dried at 40°C for 24 h, after which it was weighed. This follows our prior works [20, 21].

The color change was confirmed using a UV–Vis spectrophotometer (V‐670; Jasco, Japan) at room temperature, scanning wavelengths from 200 to 700 nm. Fourier‐transform infrared (FTIR) spectra were recorded using a Nicolet iS5 FTIR‐8400S spectrophotometer (ThermoFisher Scientific, United States) over a spectral range of 4000–400 cm^−1^ at room temperature. X‐ray diffraction (XRD) analysis was performed using a Bruker D8 Advance instrument (Bruker, Germany) equipped with Cu‐K*α* radiation (*λ* = 1.54060 Å).

The NPs were synthesized and their optical, structural, morphological, elemental, and magnetic characteristics were determined by ultraviolet‐visible spectroscopy (UV‐Vis), photoluminescence (PL), Fourier‐transform infrared spectroscopy (FTIR), X‐ray diffraction (XRD), scanning electron microscopy with energy dispersive X‐ray analysis (SEM‐EDX), transmission electron microscopy (TEM), along with characterization by vibrating sample magnetometry (VSM). The spherical NP shape was confirmed by TEM. The average particle sizes were 18.43 nm for AgNPs, 27.58 nm for Y‐AgNPS, and 26.43 nm for GCY‐AgNPS NPs [20, 21]. Our earlier research explored the eco‐friendly synthesis of NPs using extracts from pine needle leaves and reported the results of FTIR analysis, which revealed characteristic functional groups, the O▬H (phenolic/alcohol), C〓O (carbonyl), and C▬O (ether/alcohol) functional groups. In the course of NP synthesis, there was a change, specifically a shift and a decrease in intensity, in the aforementioned bands, which is a signature of the phytochemicals adsorbing onto the metal surface. This demonstrates that the bioactive compounds in the pine extract acted as both reducing and stabilizing agents in the green synthesis. Therefore, the biological effects of the green‐synthesized NPs can be ascribed to multiple bioactive compounds, rather than a single bioactive constituent [20, 21].

### 2.2. Animals

Sixty‐six healthy male Sprague–Dawley rats (*Rattus norvegicus*) with an average body weight of 180 g (aged 6 to 7 weeks) were used throughout the acclimatization and experimental periods. The animals were obtained from the Animal House, Faculty of Science, Beirut Arab University, Lebanon. Rats were housed in polypropylene cages with six rats in each cage. Animals were kept under laboratory conditions where the temperature was set at 23°C ± 2°C and the relative humidity at 55% ± 10%; a 12‐h light/dark cycle was also maintained. Standard pellets and water were provided ad libitum. All animals were acclimatized to the surrounding environment for 7 days before the experiment took place, closely tracking their health and activity during this time. All animal handling, management, and experimental design procedures were reviewed and approved by the Institutional Review Board (IRB) of the Beirut Arab University, Lebanon (IRB protocol number: 2023‐A‐0057‐S‐M‐0559). All laboratory animals were treated humanely per the National Institutes of Health guidelines (NIH).

### 2.3. Establishment of Diabetic Sprague–Dawley Rat Models

According to Satyanarayana et al. [22], the STZ used in this experiment was purchased from the Merck Company in Germany. The rats were fasted for 12 h overnight before the STZ injection, with free access to water. The rats were then given a single intraperitoneal injection of STZ dissolved in a freshly prepared 0.1 M sodium citrate buffer (pH 4.5) at a dose of 55 mg/kg body weight to induce diabetes mellitus [22]. They were allowed to drink water containing 15 g/L sucrose for 48 h, after which their blood glucose levels were measured through the tail vein using an Accu‐Chek glucometer (Contour Plus, Blood Glucose Monitoring System, Germany). To ensure that diabetes management could be initiated, a fasting blood glucose assessment was performed again 72 h post‐injection to validate that diabetes had been successfully induced. Only those animals with FBS levels ≥ 250 mg/dL were considered diabetic and taken up for further experimentation. Among the 42 rats given an STZ injection, 36 developed stable hyperglycemia, which yields an induction success rate of 86%.

### 2.4. Experimental Design

This study employed green‐synthesized silver NPs (AgNPs), yttrium‐doped silver NPs (Y‐AgNPs), and gadolinium–chromium–yttrium‐doped silver NPs (GCY‐AgNPs), all of which were prepared using pine needle leaf extract (PNLE) as delineated in Section 2.1. No mortality was recorded at the 7.5 mg/kg body weight dose following the intraperitoneal injection of various NPs. In order to ensure uniformity, all treatments were administered intraperitoneally (i.p.) each day at 10:00 a.m. In a preceding pilot study, both evaluated doses (2.5 and 7.5 mg/kg) of the NP were considered. The 7.5 mg/kg dose was chosen as it yielded the most favorable therapeutic efficacy and demonstrated no observable toxicity. For this test, 66 rats were induced with diabetes and randomly divided into 11 groups. The experiment’s design was as follows: healthy rats receiving saline served as the control group (Ctrl); healthy rats treated with pine‐needle leaf extract (PNLE, 17 *μ*g/*μ*L/g b.w.); healthy rats treated with silver NPs (AgNPs); healthy rats treated with yttrium‐doped silver NPs (Y‐AgNPs); healthy rats treated with gadolinium–chromium–yttrium‐doped silver NPs (GCY‐AgNPs); diabetic untreated rats (STZ); diabetic rats treated with silver NPs (STZ + AgNPs); diabetic rats treated with yttrium‐doped silver NPs (STZ + Y‐AgNPs); diabetic rats treated with gadolinium–chromium–yttrium‐doped silver NPs (STZ + GCY‐AgNPs); diabetic rats treated with pine‐needle leaf extract (STZ + PNLE); and diabetic rats treated with glibenclamide (STZ + Glb, 5 mg/kg), which served as the standard drug in this experiment. This study utilized intraperitoneal administration of glibenclamide for the sake of standardizing the administration route across all groups and equalizing systemic exposure, as was done in previous studies involving diabetic rat models [23].

Treatments started 4 days after the injection of STZ and were given daily for 3 weeks. Three animals from each group were dissected on the seventh day, while the remaining ones were dissected on day 21. Treatment was administered intraperitoneally for 3 weeks in turn. Blood sugar levels and weight were noted daily during the study.

### 2.5. Biochemical Analysis

The rats’ blood glucose levels were measured with a one‐touch glucometer in peripheral blood from the tail before being sacrificed. After 7 and 21 days, animals were anesthetized and sacrificed; then, blood samples were taken via cardiac puncture. After that, these samples were centrifuged at 3000 rpm for 15 min to separate serum, which was immediately transported to the laboratory for further investigation. Different biochemical parameters: The lipid profile includes measurements of total cholesterol, triglycerides (TGs), high‐density lipoprotein (HDL), low‐density lipoprotein (LDL), and very low‐density lipoprotein (VLDL), as well as liver function tests including ALT and AST. Kidney function was also evaluated by measuring serum urea, uric acid, creatinine, and albumin levels.

### 2.6. Histopathological Examination of Rat Kidney Tissues

Tissue processing was carried out using an automated tissue processor (Leica RM2235, Leica Microsystems, United States). The procedure was initiated with a two‐step fixation that included 2 cycles, each involving placing the tissue in 10% formalin for 2 h. The first step involved immersing the tissue in formalin for 2 h. The next step comprised a 30‐minute wash in distilled water to eliminate any remnants of the fixative. Subsequently, the tissue underwent an alcohol dehydration (70%, 90%, and then 100% ethanol) process, which was completed at varying time ranges. In the clearing step, the tissue was immersed in a combination (50:50) of xylene and alcohol for 30 min, rinsed in xylene for another 30 min, and then placed in xylene again for 30 min. The specimen was subsequently covered with molten wax and embedded, followed by the formation of paraffin blocks. Tissue sections were prepared at 4–5 *μ*m thickness. Finally, the slides were stained with hematoxylin and eosin (H&E). The morphological changes of the stained slides were checked under a light microscope (Leica DM500 with ICC50 W CAM, Leica Microsystems, United States). The prepared slides were then evaluated for histopathological alterations.

### 2.7. Molecular Docking Simulation

Antidiabetic protein receptors were retrieved from the Protein Data Bank and prepared by removing water molecules, ions, and bound ligands (Table S1). All crystal structures of the targets were prepared by removing water molecules, ions, and existing ligands using PyMOL software. Hydrogen atoms were then added to the receptor molecule using MG Tools of Autodock Vina software and saved in a dockable PDBQT format. The three types of silver NPs (AgNPs, Y‐AgNPs, and GCY‐AgNPs) were modeled and used as distinct ligands in the docking simulations. We used VESTA (Visualization for Electronic Structure Analysis), a 3D visualization program for structural models, volumetric data, and crystal morphologies, as follows: (1) draw the structure in CIF format (Crystallographic Information File), then the CIF format was converted to 3D Mol2 format and further converted using Open Babel to 3D PDB format. (3) The structure in PDB format was used as input for the docking simulation. (4) Internal degrees of freedom were set to zero. (5) The structure was changed to the pdbqt format using Autodock tools. (6) The chemical structures were subjected to energy minimization. Hydrogen atoms were added to both the receptors and each NP variant using Autodock Vina, with files saved in PDB format for docking simulations. Polar hydrogens and Gasteiger charges were assigned to the receptors via Autodock Tools, followed by the generation of ligand‐centered grid maps (60 × 60 × 60 Å) using AutoGrid to define the binding site. Separate docking simulations were performed for each NP type against the receptors, and the resulting complexes were analyzed in Discovery Studio 4.5 to visualize 2D hydrogen‐bond interactions between the receptors and NPs. we redocked the native small‐molecule ligands from the crystal structures using Autodock Vina and calculated the binding affinities and RMSD values relative to their experimental poses (Supplementary Table S1). RMSD values ≤ 2 Å are generally considered indicative of successful pose reproduction, confirming the reliability of our docking setup for predicting NP interactions.

### 2.8. Statistical Analysis

Group differences were evaluated with one‐way analysis of variance (ANOVA) implemented in SPSS version 24.0 (IBM, United States). Results are expressed as means ± standard error of the mean (SEM). Statistical differences with a *p* < 0.05 were considered significant.

## 3. Results

### 3.1. Clinical Observations, Body and Organ Weight, and Organ Indices

During the experiment, none of the groups showed signs of mortality. The diabetic rats (STZ group) exhibited higher food consumption, water intake, and frequent urination. On the other hand, a noticeable reduction in urination was recorded on the third day of treatment among the treated groups with different NPs.

**Figure 1:**
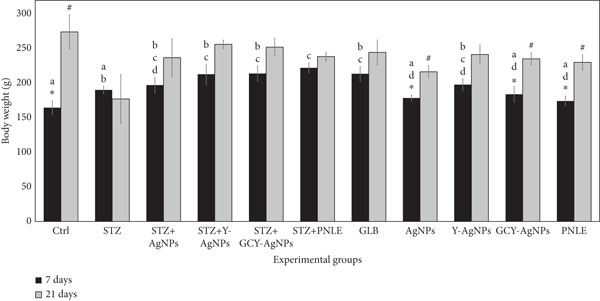
Effect of nanoparticle treatments on the body weight of diabetic rats at 7 and 21 days post‐treatment. Data are presented as mean ± standard error of the mean (SEM). Each group consisted of six rats (*n* = 6; three rats dissected at Day 7 and three at Day 21). Superscript letters (a–d) indicate within‐group differences (same letter, *p* > 0.05; different letters, *p* < 0.05), while superscript symbols (∗, #) indicate within‐time differences (same symbol, *p* > 0.05; different symbols, *p* < 0.05). A significant interaction between time and groups was detected by two‐way ANOVA (F = 2.062, *p* = 0.049).

The average body weight changes were analyzed at two time points, 7 and 21 days (Supplementary Table S2). A significant weight increase from 164.66 to 274.33 g was noted in the control group (*p* < 0.05). Among the treated groups, the AgNPs showed a significant weight gain from 178.66 to 216.66 g (*p* < 0.05), while the GCY‐AgNPs group recorded an increase from 184.00 to 235.00 g, and the PNLE group showed an increase in weight from 174.33 to 230.33 g (*p* < 0.05). On the 7th day, significant differences were observed between groups (*p* < 0.05), whereas differences on the 21st day were not statistically significant (*p* > 0.05). These findings revealed that the control group experienced a considerable weight gain, which was also notable among certain NP‐treated groups, with the PNLE showing the highest increase in weight across treatments (Figure 1). Out of all formulations of the NPs, GCY‐AgNPS demonstrated the greatest improvement in mass gain, followed by Y‐AgNPs, and then AgNPs, indicating that the level of therapeutic effect of GCY‐AgNPS was significantly greater than that of all the other treatments. Improvement in body weight in NP‐treated rats could, on the one hand, stem from regained function and restoration of the *β*‐cell in the pancreas and, on the other hand, from enhanced insulin sensitivity, which facilitates glucose disposal and the absorption of dietary constituents. In this regard, the metabolites of the pine needle extract used, particularly the phytochemicals, could also be a source of oxidative stress alleviation, with ensuing recovery of the metabolism.

Over time, organ weight and indices variations between experimental groups indicated the effects of different NP treatments on organ changes caused by STZ. By Day 7, the GLB group exhibited the highest values for liver weight (9.87 ± 0.83 g), kidney weight (2.20 ± 0.042 g), and kidney index (0.010 ± 0.0003) compared with the control group (*p* < 0.05). Among the treated groups, the STZ + PNLE and STZ + Ag exhibited increased liver weights (9.0 ± 0.10 and 9.09 ± 0.55 g, respectively, *p* < 0.05). In comparison, the GCY‐AgNPS and PNLE groups demonstrated the lowest kidney weights (1.54 ± 0.086 and 1.53 ± 0.118, respectively, *p* < 0.05), reflecting their protective effects. The decrease in hypertrophy of the liver and kidneys suggests that those NPs are lessening the oxidative stress and inflammation brought about by hyperglycemia, and thus, seem to allow the organs to function normally. The incorporation of yttrium and gadolinium may also explain the increase in the antioxidant enzymes and the enhancement of organ tissue repair, as evidenced by the organ indices. By Day 21, there was a noticeable increase in kidney weight for the STZ group, indicating sustained hypertrophy (2.69 ± 0.19 g) and index (0.016 ± 0.002) as opposed to other treated groups such as GLB, Ag, and PNLE, which significantly reduced their respective kidney index values (0.008 ± 0.0002, 0.007 ± 0.00006, and 0.007 ± 0.00007, respectively, *p* < 0.05), approaching control levels. Notably, the STZ + GCY‐AgNPS group exhibited significant improvement in liver weight (7.81 ± 0.19 to 9.75 ± 0.44 g, *p* < 0.05) and kidney weight (1.54 ± 0.086 to 1.81 ± 0.01 g, *p* < 0.05) over time, respectively (Table 1).

**Table 1:** Variation in organ weights and indices among experimental groups at different time intervals.

**Parameters**	**Time (days)**	**Experimental groups**											**Two-way ANOVA**
		**Ctrl**	**STZ**	**STZ + AgNPs**	**STZ + Y-AgNPS**	**STZ + GCY-AgNPS**	**STZ + PNLE**	**GLB**	**AgNPs**	**Y-AgNPS**	**GCY-AgNPS**	**PNLE**	
Liver weight (g)	7	6.7 ± 0.17^a^	7.7 ± 0.21^ab^	9.09 ± 0.55^bc^	8.79 ± 0.85^bcd^	7.81 ± 0.19^ab,^^∗^	9.0 ± 0.10^bc^	9.87 ± 0.83^c^	7.90 ± 0.11^ab^	8.11 ± 0.48^ab^	7.35 ± 0.68^ad^	6.78 ± 0.37^a^	F = 1.850*p* = 0.079
	21	9.89 ± 1.71	8.92 ± 0.70	8.42 ± 0.90	10.26 ± 0.62	9.75 ± 0.44^#^	8.98 ± 0.79	8.66 ± 0.62	7.07 ± 0.50	8.21 ± 0.49	8.81 ± 0.27	8.46 ± 1.31	
Liver index	7	0.041 ± 0.002	0.041 ± 0.001	0.046 ± 0.005	0.041 ± 0.001	0.036 ± 0.0009	0.040 ± 0.0009	0.046 ± 0.002^∗^	0.044 ± 0.0009^∗^	0.041 ± 0.002	0.039 ± 0.001	0.038 ± 0.0006	F = 1.217 *p* = 0.034
	21	0.035 ± 0.003	0.054 ± 0.011	0.035 ± 0.0003	0.040 ± 0.003	0.038 ± 0.001	0.037 ± 0.003	0.035 ± 0.002^#^	0.032 ± 0.002^#^	0.034 ± 0.0008	0.037 ± 0.001	0.036 ± 0.004	
Kidney weight (g)	7	1.39 ± 0.018^a,^^∗^	1.93 ± 0.034^bcd,^^∗^	2.08 ± 0.171^cd^	1.87 ± 0.184^bc^	1.64 ± 0.095^ab^	1.91 ± 0.127^bcd^	2.20 ± 0.042^d^	1.72 ± 0.010^be^	1.63 ± 0.119^ab^	1.54 ± 0.086^ae,^^∗^	1.53 ± 0.118^ae^	F = 2.781 *p* = 0.009
	21	2.07 ± 0.19^ac,#^	2.69 ± 0.19^b,#^	1.80 ± 0.31^ac^	2.10 ± 0.09^a^	1.99 ± 0.14^ac^	2.01 ± 0.07^ac^	2.08 ± 0.19^ac^	1.62 ± 0.05^c^	1.73 ± 0.11^ac^	1.81 ± 0.01^ac,#^	1.75 ± 0.07^ac^	
Kidney index	7	0.008 ± 0.0004^ae^	0.010 ± 0.0004^bcd^	0.010 ± 0.0015^c^	0.008 ± 0.0002^abe^	0.007 ± 0.00006^e^	0.008 ± 0.0005^abe^	0.010 ± 0.0003^dc,^^∗^	0.009 ± 0.0002^abcd,^^∗^	0.008 ± 0.0002^ae,^^∗^	0.008 ± 0.00008^ae^	0.008 ± 0.0003^abde,^^∗^	F = 4.818 *p* = 0.001
	21	0.007 ± 0.00007^a^	0.016 ± 0.002^b^	0.007 ± 0.0004^a^	0.008 ± 0.0002^a^	0.007 ± 0.0001^a^	0.008 ± 0.0004^a^	0.008 ± 0.0002^a,#^	0.007 ± 0.00006^a,#^	0.007 ± 0.0001^a,#^	0.007 ± 0.0003^a^	0.007 ± 0.00007^a,#^	

*Note:* Data are presented as mean ± standard error of the mean (SEM). Superscript letters (a–e) denote within‐group comparisons (same letter, *p* > 0.05; different letters, *p* < 0.05), while superscript symbols (∗, #) denote comparisons between time intervals (same symbol, *p* > 0.05; different symbols, *p* < 0.05). Each group consisted of six rats (*n* = 6; three rats dissected at Day 7 and three at Day 21).

### 3.2. Effect of NPs on Fasting Blood Glucose Level

**Figure 2:**
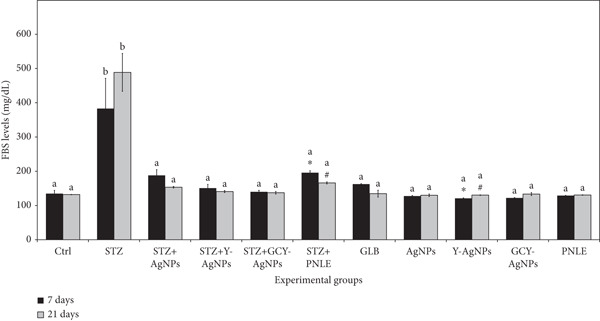
Fasting blood glucose levels in diabetic rats treated with various nanoparticles following 7 and 21 days of treatment. Data are presented as mean ± standard error of the mean (SEM), with error bars indicating SEM. Each group consisted of six rats (*n* = 6; three rats dissected at Day 7 and three at Day 21). Superscript letters (a, b) denote within‐group comparisons (same letter, *p* > 0.05; different letters, *p* < 0.05), while superscript symbols (∗, #) denote within‐time comparisons (same symbol, *p* > 0.05; different symbols, *p* < 0.05). A significant interaction between time and groups was observed by two‐way ANOVA (F = 1.363, *p* > 0.05).

Serum FBS levels showed distinct differences between the experimental groups at both intervals (Figure 2). On the 7th day, the STZ group indicated an increased FBS level of 383.00 mg/dL, significantly greater than the control group (134.66 mg/dL). Among the treated groups, the lowest FBS (140.00 mg/dL) was recorded by the STZ + GCY‐AgNPS group, while the STZ + Y‐AgNPS group followed with 150.66 mg/dL. By Day 21, the STZ group experienced a further elevation in FBS concentrations to 488.66 mg/dL, which indicated persistent hyperglycemia. In contrast, all other treated groups showed improvement in fasting blood glucose levels, particularly STZ + GCY‐AgNPS, with a value of 137.33 mg/dL. The greatest decrease in FBS over time was recorded in the STZ + PNLE group, which markedly decreased from 195.66 mg/dL on Day 7 to 166.00 mg/dL on Day 21 (*p* < 0.05). The reduction observed in this PNLE‐treated group can be explained by the presence of pine needle leaf extract’s polyphenols and flavonoids, which have antioxidant and insulin‐sensitizing properties that facilitate glucose metabolism and alleviate oxidative stress in diabetic rats.

The treated groups’ blood glucose values being lower indicates the recovery of pancreatic *β*‐cell functioning and insulin secretion, along with injury from glucose‐STZ oxidative damage, and the ability to facilitate glucose uptake is also preserved. The doped NPs (GCY‐AgNPs) probably exhibited additional mitigative and antioxidant effects against the reactive oxygen species attacking insulin‐producing pancreatic cells. Moreover, additional glycemic control improvement also came from the insulin‐sensitizing, antioxidant, and anti‐inflammatory effects of the pine needle extract.

### 3.3. Effect of NP Administration on Biochemical Parameters

#### 3.3.1. Effect of NPs on Liver Function Tests

There were some differences in AST and ALT serum concentrations among the groups under consideration for both time points (Figure 3). On Day 7, the STZ group exhibited the highest ALT (132.66 IU/L) and AST (731.00 IU/L) levels, indicating more severe liver damage in the STZ group compared with the controls. The STZ + GCY‐AgNPS group, on the other hand, demonstrated the lowest levels of ALT (19.33 IU/L, *p* < 0.05) and AST (109.33 IU/L, *p* < 0.05), making it the most effective group for mitigating liver impairment. By Day 21, the STZ group experienced further elevation in ALT levels (180.00 IU/L) and sustained high AST levels (471.33 IU/L). In contrast, the STZ + GCY‐AgNPS group maintained ALT and AST levels statistically significant from the onset, recorded at 25.33 IU/L and 147.33 IU/L, respectively (*p* < 0.05). By the end of the 21 days, it was noted that the STZ + Ag group considerably decreased their ALT (29.33 IU/L, *p* < 0.05) and AST (182.66 IU/L, *p* < 0.05) levels.

Figure 3Levels of hepatic functional markers (a) aspartate aminotransferase and (b) alanine aminotransferase in the serum of the diabetic rats and rats treated with various nanoparticles measured at 7 and 21 days. Data are presented as mean ± standard error of the mean (SEM), with error bars indicating SEM. Each group consisted of six rats (*n* = 6; three rats dissected at Day 7 and three at Day 21). Superscript letters (a–d) denote within‐group comparisons (same letter, *p* > 0.05; different letters, *p* < 0.05), while superscript symbols (∗, #) denote within‐time comparisons (same symbol, *p* > 0.05; different symbols, *p* < 0.05). Significant interactions in AST and ALT levels between time and groups were observed by two‐way ANOVA (F = 4.109, *p* < 0.001; and F = 14.327, *p* < 0.001, respectively).

**Figure:**
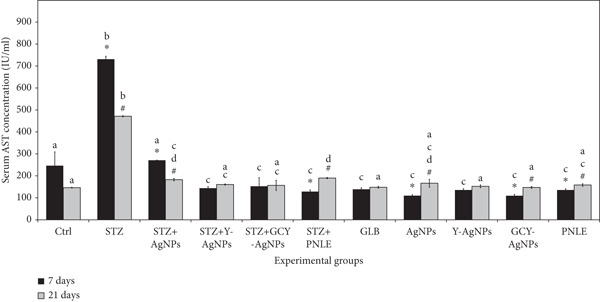
(a) Serum AST Concentration (IU/mL)s

**Figure:**
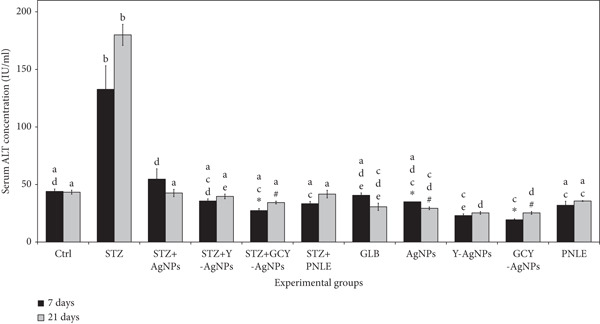
(b) Serum ALT Concentration (IU/mL)

Reduced levels of AST and ALT enzymes within groups treated with doped silver NPs and pine needle extracts showcase the hepatoprotective potential of these NPs and the impact of possible alleviation of oxidative stress and inflammation after STZ toxicity. Apart from the enzymatic release from the hepatocellular circulation, the doped silver NPs and pine needle extracts could have stabilized the hepatocyte membranes. Particularly, Y‐ and Gd‐doping could explain the enhancement of the silver NPs’ ROS‐scavenging potential, thus rehabilitating liver tissue and permitting the enzymes in the liver to return to normal levels, which explains the decreased serum liver enzymes.

#### 3.3.2. Effect of NPs on Kidney Function Tests

The measured concentrations of serum creatinine, urea, and uric acid showed some variations between experimental groups, reflecting the renal effects of the treatments (Figure 4). On Day 7, the STZ group revealed the highest levels of creatinine, urea, and uric acid (0.26, 63.43, and 3.56 mg/dL), respectively, which reflects marked renal damage compared with the control. However, in terms of treatment, the Ag group showed the lowest creatinine level at 0.20 mg/dL (*p* < 0.05), as well as the least amount of urea (24.00 mg/dL, *p* < 0.05) and uric acid (2.23 mg/dL), indicating potential for strong tissue repair activity. The STZ group exhibited an increase in creatinine (1.00 mg/dL, *p* < 0.05) and uric acid (5.00 mg/dL) on Day 21 of the experiment. In comparison, urea levels decreased moderately but remained high (58.33 mg/dL). On the other hand, there were improvements in treated groups; for instance, STZ + GCY‐AgNPS exhibited low levels of creatinine (0.40 mg/dL), with a highly reduced amount of urea to around 34.66 mg/dL and a drop in uric acid to 2.56 mg/dL, followed by the STZ + Y‐AgNPS group. Moreover, the STZ + Ag group exhibits a significantly low level of renal functional markers (*p* < 0.05). In addition, the GLB group reduced uric acid concentration from 3.03 mg/dL on Day 7 to 2.33 mg/dL on Day 21 (*p* < 0.05).

Figure 4Serum levels of renal functional markers (a) creatinine, (b) urea, and (c) uric acid in diabetic rats and rats treated with various nanoparticles at 7 and 21 days post‐treatment. Data are presented as mean ± standard error of the mean (SEM), with error bars indicating SEM. Each group consisted of six rats (*n* = 6; three rats dissected at Day 7 and three at Day 21). Superscript letters (a–d) denote within‐group comparisons (same letter, *p* > 0.05; different letters, *p* < 0.05), while superscript symbols (∗, #) denote within‐time comparisons (same symbol, *p* > 0.05; different symbols, *p* < 0.05). A significant interaction was observed in creatinine levels between time and groups by two‐way ANOVA (F = 13.092, *p* < 0.001). No significant interactions were observed for urea or uric acid (F = 0.549, *p* > 0.05; and F = 1.823, *p* > 0.05, respectively).

**Figure:**
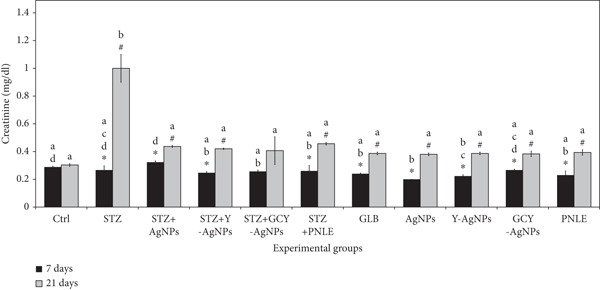
(a)

**Figure:**
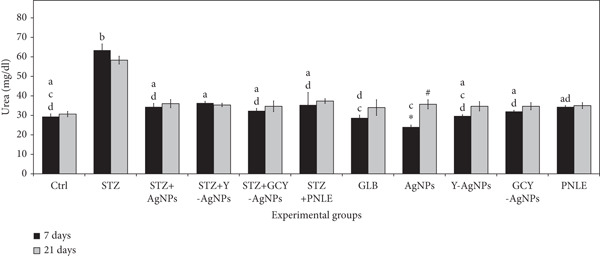
(b)

**Figure:**
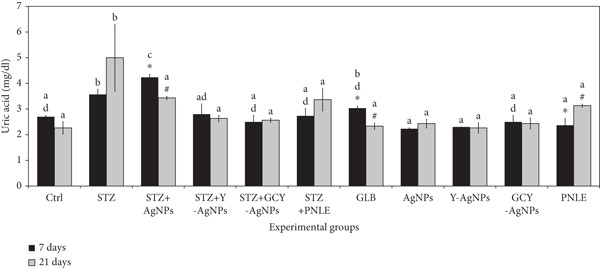
(c)

The changes in the renal biomarkers suggest that the NPs diminished the oxidative damage and inflammation in the kidneys following diabetes induction by STZ. The antioxidative and free radical–scavenging activities of Ag, Y, and Gd–Cr–Y doped NPs could have shielded the glomeruli and renal tubules from the damaging effects of oxidation, thereby minimizing the blood urea nitrogen and creatinine levels. Furthermore, the constituents of the pine needles used in conjunction with the study may have also assisted in renal recovery through the modulation of the renal antioxidant system and the enhancement of glomerular filtration, as previously described.

### 3.4. Effect of NPs on Lipid Profile

Changes in cholesterol, TGs, HDL, and LDL concentration varied significantly across the experimental groups over time, as detailed in Table 2. On Day 7, the GLB group exhibited the highest concentration of cholesterol (66.66 ± 0.66 mg/dL, *p* < 0.05), while the STZ + Y‐AgNPS group showed the lowest concentration (49.66 ± 4.80 mg/dL). By Day 21, the GLB group demonstrated a marked reduction in cholesterol levels, comparable with the control (49.66 ± 2.02 mg/dL, *p* < 0.05). In contrast, the STZ + GCY‐AgNPS group indicates a significant cholesterol rise, indicating lowered effectiveness. The STZ group exhibited the highest values of TGs on the 7th day (56.33 ± 2.33 mg/dL, *p* < 0.05) while the PNLE group had the lowest amount at (31.00 ± 1.52 mg/dL, *p* < 0.05). Furthermore, on Day 21, the STZ group experienced an increase in the value of (86.66 ± 3.28 mg/dL, *p* < 0.05). In contrast, the STZ + PNLE group exhibited variability between groups, but the average value was reported at a rise of (60.33 ± 2.33 mg/dL, *p* < 0.05). It is worth noticing that the 7‐day average was the highest for HDL at (43.66 ± 1.45 mg/dL, *p* < 0.05), but on the 21‐day, the most significant increase was produced by the GLB group at (49.33 ± 0.66 mg/dL, *p* < 0.05). The STZ group experienced a drop in the HDL level (28.66 ± 0.88 mg/dL, *p* < 0.05), indicating lipid profile deterioration. The highest level of LDL was found for the STZ + Ag group (12.00 ± 1.00 mg/dL), while the STZ + Y‐AgNPS group exhibited the lowest level (7.33 ± 0.66 mg/dL, *p* < 0.05) on Day 7. However, on Day 21, the STZ + GCY‐AgNPS group experienced the most significant reduction in LDL (9.10 ± 0.10 mg/dL) compared with the diabetic STZ group (13.33 ± 0.88 mg/dL, *p* < 0.05).

**Table 2:** Variations in lipid profile parameters across experimental groups over time.

**Parameters**	**Time (days)**	**Experimental groups**											**Two-way ANOVA**
		**Ctrl**	**STZ**	**STZ + AgNPs**	**STZ + Y-AgNPS**	**STZ + GCY-AgNPS**	**STZ + PNLE**	**GLB**	**AgNPs**	**Y-AgNPS**	**GCY-AgNPS**	**PNLE**	
Cholesterol (mg/dL)	7	51.66 ± 1.45^a^	77.33 ± 8.81^b^	71.00 ± 3.51^cb^	49.66 ± 4.80^a^	55.33 ± 2.33^ad^	62.33 ± 4.91^ac^	66.66 ± 0.66^bcd,^^∗^	60.66 ± 2.96^ac^	56.33 ± 0.33^ad^	54.33 ± 2.66^ad,^^∗^	61.33 ± 7.88^acd^	F = 3.659 *p* = 0.001
	21	49.66 ± 2.60^a^	72.00 ± 2.64^b^	69.00 ± 1.73^b^	54.66 ± 0.88^acd^	52.33 ± 2.18^ac^	57.66 ± 1.33^cd^	49.66 ± 2.02^a,#^	55.33 ± 1.20^cd^	71.66 ± 1.85^b^	69.33 ± 2.33^b,#^	58.00 ± 1.15^d^	
Triglyceride (mg/dL)	7	40.33 ± 2.33^ac^	56.33 ± 2.33^b,^^∗^	48.66 ± 0.88^ab,^^∗^	45.33 ± 3.17^ab,^^∗^	48.66 ± 0.33^ab,^^∗^	31.00 ± 1.52^c,^^∗^	39.00 ± 0.000^ac,^^∗^	35.66 ± 7.83^ac^	39.66 ± 2.18^ac^	54.66 ± 12.12^b^	27.00 ± 2.51^c^	F = 5.506 *p* = 0.001
	21	41.33 ± 2.90^ad^	86.66 ± 3.28^b,#^	59.33 ± 1.20^c,#^	56.33 ± 1.20^c,#^	54.33 ± 1.20^c,#^	60.33 ± 2.33^c,#^	53.33 ± 1.76^c,#^	43.66 ± 4.66^d^	40.66 ± 5.66^ad^	38.33 ± 2.02^ad^	34.33 ± 1.76^a^	
HDL (mg/dL)	7	33.66 ± 2.40^ac^	42.00 ± 0.000^bd,^^∗^	36.00 ± 3.05^ae^	29.33 ± 2.60^c^	37.00 ± 2.08^abe^	39.33 ± 0.33^abd^	43.66 ± 1.45^d,^^∗^	42.33 ± 2.33^bd^	38.00 ± 2.08^abde,^^∗^	32.33 ± 2.02^ce,^^∗^	37.33 ± 0.88^abe,^^∗^	F = 8.391 *p* = 0.001
	21	33.00 ± 3.60^ae^	28.66 ± 0.88^a,#^	39.66 ± 1.85^bde^	32.00 ± 1.52^a^	34.66 ± 0.33^ab^	30.33 ± 5.23^a^	34.66 ± 0.88^ab,#^	43.00 ± 1.52^cd^	48.33 ± 2.72^c,#^	49.33 ± 0.66^c,#^	41.00 ± 0.57^bd,#^	
LDL (mg/dL)	7	9.00 ± 0.00	10.33 ± 2.02	12.00 ± 1.00	7.33 ± 0.66^∗^	9.00 ± 0.57	7.66 ± 1.76	8.33 ± 0.33	8.00 ± 0.57	9.33 ± 1.20	11.00 ± 1.52	8.00 ± 0.000	F = 1.434 *p* = 0.197
	21	8.33 ± 0.66^ac^	13.33 ± 0.88^b^	10.33 ± 1.33^ab^	9.66 ± 0.33^ac,#^	9.10 ± 0.10^ac^	10.66 ± 0.33^ab^	8.50 ± 0.28^ac^	8.33 ± 1.85^ac^	11.00 ± 0.57^ab^	9.00 ± 1.52^ac^	6.66 ± 1.66^c^	

*Note:* Data are presented as mean ± standard error of the mean (SEM). Superscript letters (a–e) denote within‐group comparisons (same letter, *p* > 0.05; different letters, *p* < 0.05), while superscript symbols (∗, #) denote comparisons between time intervals (same symbol, *p* > 0.05; different symbols, *p* < 0.05). Each group consisted of six rats (*n* = 6; three rats dissected at Day 7 and three at Day 21).

The low values of the lipid profile parameters recorded in the rats treated with the nanocarrier suggest that the impacted the rats’ lipid metabolism through the mechanisms of insulin sensitivity and the reduction of liver oxidative stress in the treated rats. There may also be antioxidant and metal‐chelating activity of the doped NPs that prevented lipid peroxidation and normalized the concentration of HMG‐CoA reductase and the other enzymes of cholesterol and TG biosynthesis, which would explain the reduction in lipid synthesis. Furthermore, the bioactive constituents of the pine needle extract would have contributed to the maintenance of lipid homeostasis in view of their anti‐inflammatory and lipid‐lowering effects.

### 3.5. Effect of NPs on Serum Glucose and Albumin

The variation in serum glucose and albumin concentrations among experimental groups revealed significant differences over time, highlighting the impact of various treatments (Table 3). On Day 7, the STZ + PNLE group had significantly higher glucose levels (71.33 ± 2.40 mg/dL, *p* < 0.05) compared with the control group (45.33 ± 2.33 mg/dL). In comparison, STZ + Ag and STZ + GCY‐AgNPS groups showed relatively lower glucose levels (51.33 ± 3.48 and 59.33 ± 6.93 mg/dL, respectively), which indicates more effective glycemic control in these groups. By Day 21, the serum glucose levels of STZ + Ag and STZ + Y‐AgNPS groups dropped to levels that were not significantly different from the control group (124.66 ± 0.88 and 124.33 ± 2.18 mg/dL, respectively, *p* < 0.05). However, the STZ group still had significantly higher levels (286.33 ± 25.88 mg/dL), which indicates hyperglycemia (untreated). On Day 7, the STZ + Y‐AgNPS group exhibited a significantly lower albumin level (3.33 ± 0.08 mg/dL, *p* < 0.05) compared with the control (4.10 ± 0.05 mg/dL). There was a significant improvement by Day 21 in the albumin levels of STZ + Ag and STZ + Y‐AgNPS groups. The mean values for STZ + Ag and STZ + Y‐AgNPS groups were (4.10 ± 0.05 and 3.86 ± 0.06 mg/dL, respectively, at *p* < 0.05), close to the control group values. Conversely, the STZ + GCY‐AgNPS group lowered glycemic control from 3.60 ± 0.00 mg/dL on Day 7 to 3.36 ± 0.18 mg/dL on Day 21. The analysis of the data generated revealed that the groups differed significantly, as shown by the two‐way ANOVA (*p* < 0.05), where the STZ + Ag group showed the most significant improvement in glycemic control over time (*p* < 0.001), followed by STZ + GCY‐AgNPS (*p* = 0.001).

**Table 3:** Changes in glucose and albumin levels between experimental groups over time intervals.

**Parameters**	**Time (days)**	**Experimental groups**											**Two-way ANOVA**
		**Ctrl**	**STZ**	**STZ + AgNPs**	**STZ + Y-AgNPS**	**STZ + GCY-AgNPS**	**STZ + PNLE**	**GLB**	**AgNPs**	**Y-AgNPS**	**GCY-AgNPS**	**PNLE**	
Glucose (mg/dl)	7	45.33 ± 2.33^a,^^∗^	66.00 ± 4.35^be,^^∗^	51.33 ± 3.48^ad,^^∗^	65.66 ± 6.00^be,^^∗^	59.33 ± 6.93^bd,^^∗^	71.33 ± 2.40^e,^^∗^	59.00 ± 3.21^bd,^^∗^	99.00 ± 1.73^c,^^∗^	80.66 ± 1.45^fe,^^∗^	78.33 ± 2.33^fe,^^∗^	83.33 ± 1.76^f,^^∗^	F = 20.168 *p* = 0.001
	21	123.33 ± 22.16^a,#^	286.33 ± 25.88^b,#^	124.66 ± 0.88^a,#^	124.33 ± 2.18^a,#^	123.00 ± 1.52^a,#^	143.33 ± 8.41^a,#^	123.33 ± 1.66^a,#^	136.66 ± 6.98^a,#^	124.33 ± 0.66^a,#^	122.66 ± 1.76^a,#^	117.66 ± 0.33^a,#^	
Albumin (mg/dl)	7	4.10 ± 0.05^a^	4.10 ± 0.000^a^	3.76 ± 0.06^bd,^^∗^	3.33 ± 0.08^e^	3.60 ± 0.000^bc^	4.13 ± 0.14^a^	4.03 ± 0.03^a^	3.93 ± 0.03^ad^	3.53 ± 0.08^bce,^^∗^	3.46 ± 0.20^ce^	3.76 ± 0.03^bd^	F = 1.826 *p* = 0.084
	21	4.13 ± 0.06^a^	4.20 ± 0.15^a^	4.10 ± 0.05^ac,#^	3.63 ± 0.13^bd^	3.36 ± 0.18^d^	4.20 ± 0.11^a^	3.93 ± 0.17^ab^	3.66 ± 0.20^bcd^	3.86 ± 0.06^ab,#^	3.93 ± 0.08^ab^	3.96 ± 0.28^ab^	

*Note:* Data are presented as mean ± standard error of the mean (SEM). Superscript letters (a–f) denote within‐group comparisons (same letter, *p* > 0.05; different letters, *p* < 0.05), while superscript symbols (*, #) denote comparisons between time intervals (same symbol, *p* > 0.05; different symbols, *p* < 0.05). Each group consisted of six rats (*n* = 6; three rats dissected at Day 7 and three at Day 21).

NP treatment signifies the restoration of glucose homeostasis and glucose homeostasis via enhancement of insulin secretion and reduction of oxidative stress. This is evidenced by the decrease in serum glucose and the increase in serum albumin levels. Hepatic protein synthesis is also restored, as indicated by the improvement in albumin levels. The AgNPs, Y‐AgNPs, and GCY‐AgNPs’ potential protective effect on hepatocytes and pancreatic *β*‐cells from STZ‐induced damage is possibly a result of their potent anti‐oxidative and anti‐inflammatory properties. Improvement in albumin levels suggests that the green‐synthesized NPs reduced hepatic injury due to their stabilizing effects and thus maintained the hepatic synthetic function.

### 3.6. Histopathological Findings

**Figure 5:**
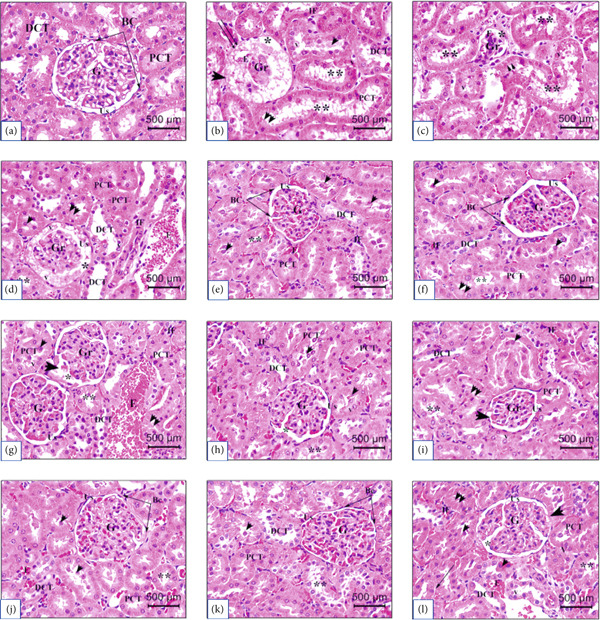
Histological alterations in the kidney tissues of control and treated groups after 21 days. Representative photomicrographs of serial kidney tissue sections from rats in the different experimental groups: (a) Ctrl, normal control; (b, c) STZ, Streptozotocin‐induced diabetic; (d) STZ + Ag, STZ‐Diabetic rats treated with Ag NPs; (e) STZ + Y‐AgNPS, STZ‐Diabetic rats treated with Y‐AgNPS NPs; (f) STZ + GCY‐AgNPS, STZ‐Diabetic rats treated with GCY‐AgNPS NPs; (g) STZ + PNLE, STZ + GCY‐AgNPS, STZ‐Diabetic rats treated with pine needle leaf extract; (h) STZ + Glb, STZ + GCY‐AgNPS, STZ‐Diabetic rats treated with glibenclamide; (i) Ag, Ag NPs‐treated; (j) Y‐AgNPS, Y‐AgNPS NPs‐treated; (k) GCY‐AgNPS, GCY‐AgNPS NPs‐treated; (l) PNLE, pine needle leaf extract‐treated. Representative H&E‐stained kidney sections (400×; scale bar = 500 *μ*m. Normal renal structures are shown, including glomerulus (G), urinary space (Us), Bowman’s capsule lined by squamous cells (BC), proximal convoluted tubule (PCT) with brush border, and distal convoluted tubule (DCT). Atrophied renal corpuscle (double arrow), with degenerated and reduced glomerulus (Gr) congested with erythrocytes (E), dilatation of the Us (∗), desquamated BC (large arrowhead), peritubular infiltration (IF), damaged PCT with cellular shedding (arrowhead), irregular cellular arrangement (double arrowhead), and loss of brush borders (∗∗), cellular vacuolization (V), detachment of PCT cells from basement membrane (arrow). Each group consisted of six rats (*n* = 6; three rats dissected at Day 7 and three at Day 21).

The effects of the intraperitoneal administration (7.5 mg/kg) of the tested green synthesized NP alone and against STZ‐induced diabetes in rats’ toxicities were examined after 21 days of treatment. Most organs exhibited no significant histopathological alterations when compared with controls. Figure 5a shows H&E‐stained kidney sections from the control (Ctrl) group after 21 days. In contrast, intraperitoneal injection of STZ (55 mg/kg) induced several renal pathological alterations compared with the untreated Ctrl group. After 21 days of STZ administration, severe pathological features in the renal cortical area were detected, including atrophied renal corpuscles with marked congested and shrunken glomeruli that exhibited signs of degeneration and atrophy at certain foci (Figure 5b,c). Besides, the Bowman’s capsule appeared damaged with a desquamated epithelium, with a severe widening and dilatation of the urinary space. In contrast, the proximal convoluted tubules (PCT) showed necrotic changes characterized by distortion of the tubular architecture and loss of the brush border of PCT cells, where most PCT cells appeared hypertrophied with cytoplasmic vacuolization and pyknotic nuclei. In addition, numerous PCT cells exhibited cellular detachment from the basement membrane and cellular shedding in the PCT lumen. Besides, increased peritubular inflammatory cell infiltration and congestion were manifested among several renal corpuscles and tubules. However, distal convoluted tubules (DCT) revealed minimal alteration in the tubular architecture.

However, STZ + Ag administration displayed mild histopathological features compared with their corresponding observed STZ group (Figure 5d). The changes were manifested with minimal alteration in the renal cortical and tubular architecture, including a slight reduction in glomeruli, minimal dilatation of the urinary space, a few irregularities of renal tubule morphology, moderate alteration in PCT cellular arrangement with minimal cytoplasmic vacuolization, and cellular shedding within the lumen. Besides, peritubular spaces showed high erythrocyte congestion and were slightly occupied with a few inflammatory cell infiltrations. DCT showed an almost normal appearance. On the other hand, H&E‐stained kidney sections of STZ + Y‐AgNPS (Figure 5e) and STZ + GCY‐AgNPS (Figure 5f) groups exhibited minimal histological alterations and revealed a protective effect, respectively, against STZ administration. In addition, GCY‐AgNPS intraperitoneal treatment after STZ administration revealed normal renal cortical and tubular architecture that greatly resembles the normal histology observed in the Ctrl group, though minimal and insignificant pathological features were detected in minimal foci. Nevertheless, the STZ + PNLE (Figure 5g) treatment group attained reasonable and additional histopathological alterations compared with their corresponding STZ + Ag, STZ + Y‐AgNPS, and STZ + GCY‐AgNPS groups. Yet, such changes were minimally manifested in correspondence to those observed in the STZ group. The most common histological alterations included a reduction in the glomerular dimensions, a few necrotic PCT with evident loss of brush border, as well as extensive congestion and inflammatory cell infiltration among the PCT peritubular spaces. Moreover, the administration of Glb as a positive control treatment against STZ‐induced diabetic rats was highly demonstrated in its corresponding H&E‐stained histological slides (Figure 5h), where the STZ + Glb group attained a regular histological appearance with renal cortical and tubular architectural preservation that were comparable with those observed in STZ + GCY‐AgNPS, as well as nearly similar to those recorded in the Ctrl group. The histopathological findings providing evidence for the protective and restorative effects on renal tissues for AgNP‐, Y‐AgNPS‐, and GCY‐AgNPS‐treated groups must primarily be attributed to the mitigation of STZ‐induced oxidative stress through the antioxidants and anti‐inflammatories. Such effects may have involved the stabilization of the renal cell membranes and the alleviation of lipid peroxidation, which may have aided the restoration of the renal tissues. The GCY‐AgNPS group yielded the best results, which suggests that synergistic dopant activity results in efficient free radical scavenging as well as restoration of the renal structure to normal.

Alternatively, H&E‐stained histological kidney sections Ag (Figure 5i), Y‐AgNPS (Figure 5j), and GCY‐AgNPS (Figure 5k) groups showed gradual preservation of normal kidney histological architecture, respectively, when compared with those observed in the Ctrl group, where most glomeruli, renal corpuscles, PCT, and DCT revealed normal appearance. Besides, mild to minimal insignificant histological changes were detected in minimal foci, where few PCTs showed minimal loss of brush border as well as irregularities in the cellular arrangement. In contrast, PNLE administration alone (Figure 5l) displayed and retained minor to reasonable histopathological features when compared with those recorded in the Ctrl group as well as to Ag, Y‐AgNPS, and GCY‐AgNPS groups, including desquamated Bowman’s capsule at minimal foci, irregular PCT with minimal loss of brush borders, cellular vacuolization, as well as PCT cellular detachment from the basement membrane.

### 3.7. Molecular Docking of AgNPs, Y‐AgNPS, and GCY‐AgNPS With Antidiabetic Target Proteins

**Figure 6:**
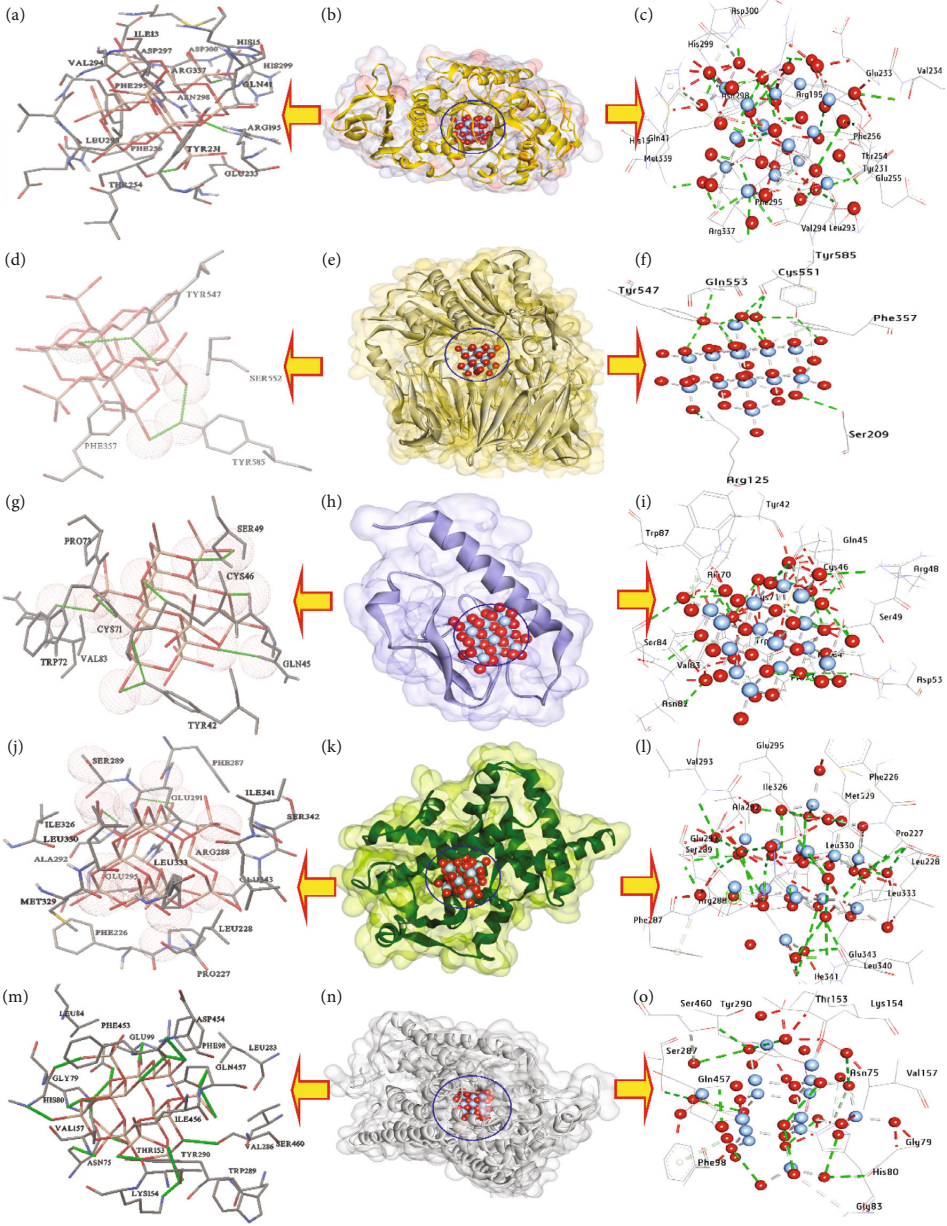
3D interactions of AgNPs conformations at the binding pocket: (a, b, and c) Amylase (PDB: ID 5U3A), (d, e, and f) DPP4 (PDB: ID 4A5S), (g, h, and i) GLP‐1R (PDB: ID 4ZGM), (j, k, and l) PPAR*γ* (PDB: ID 9F7W), (m, n, and o) SGLT2 (PDB: ID 8HEZ).

**Figure 7:**
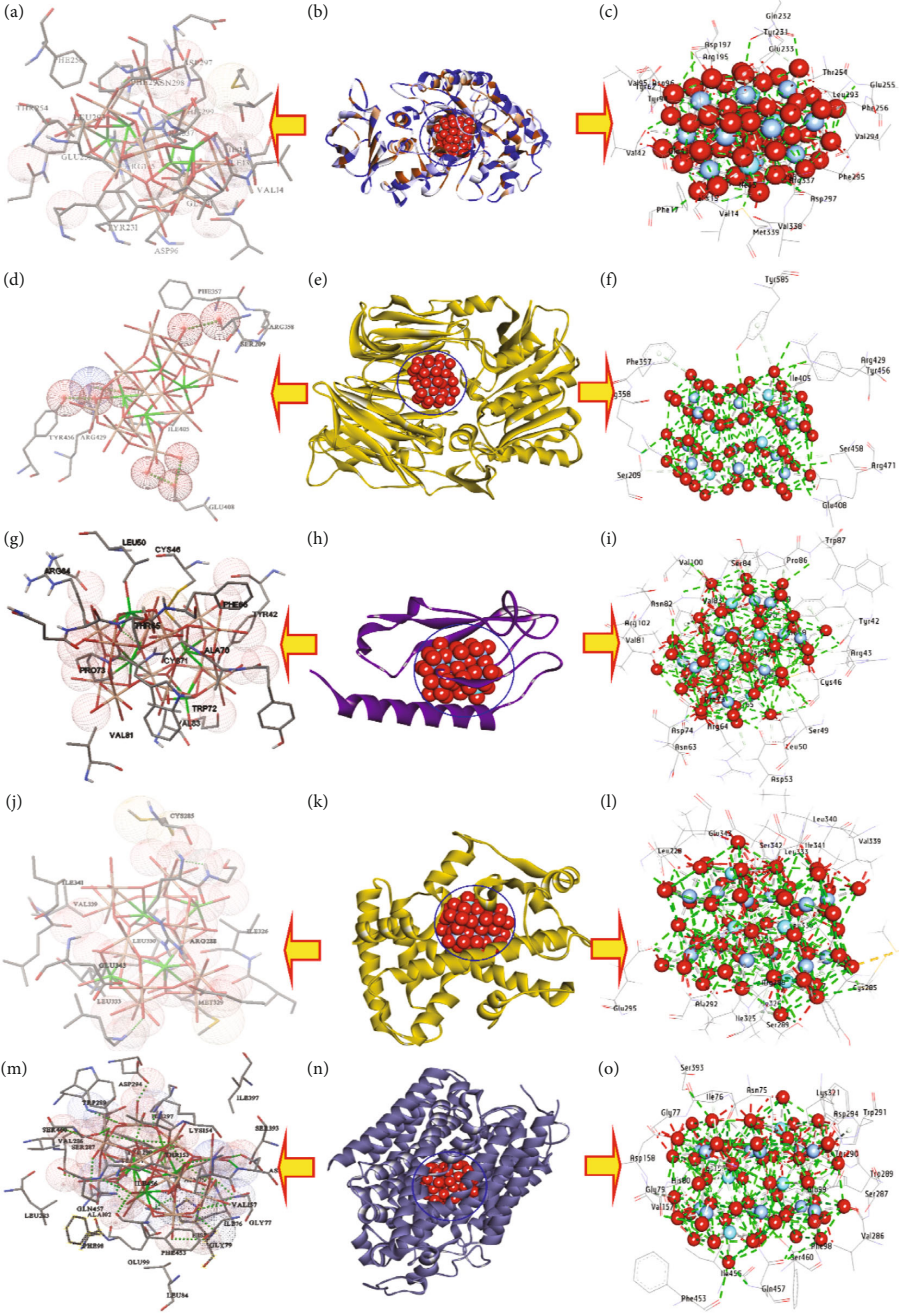
3D interactions of Y‐AgNPS conformations at the binding pocket: (a, b, and c) Amylase (PDB: ID 5U3A), (d, e, and f) DPP4 (PDB: ID 4A5S), (g, h, and i) GLP‐1R (PDB: ID 4ZGM), (j, k, and l) PPAR*γ* (PDB: ID 9F7W), (m, n, and o) SGLT2 (PDB: ID 8HEZ).

**Figure 8:**
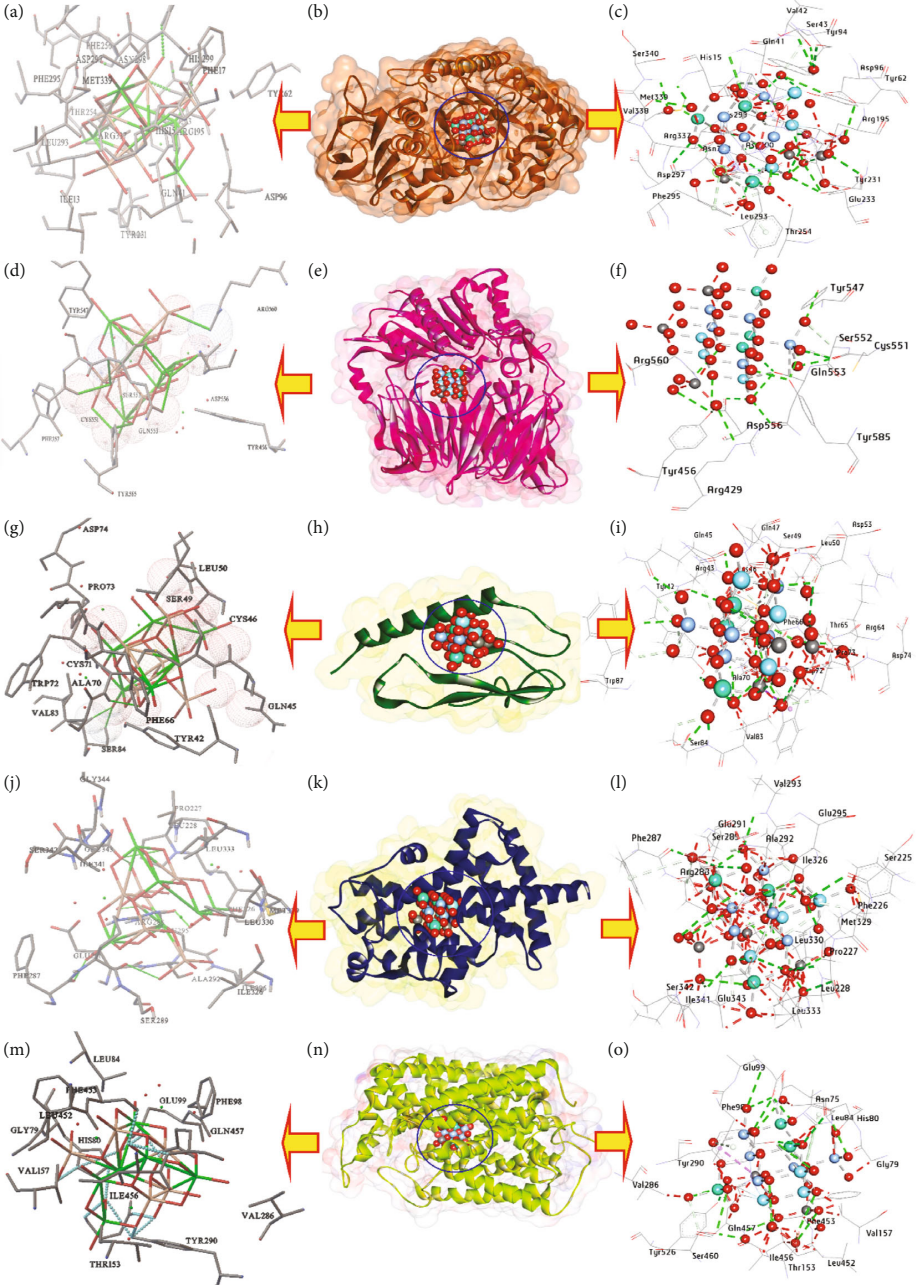
3D interactions of GCY‐AgNPS conformations at the binding pocket: (a, b, and c) Amylase (PDB: ID 5U3A), (d, e, and f) DPP4 (PDB: ID 4A5S), (g, h, and i) GLP‐1R (PDB: ID 4ZGM), (j, k, and l) PPAR*γ* (PDB: ID 9F7W), (m, n, and o) SGLT2 (PDB: ID 8HEZ).

The study on silver NPs (AgNPs, Y‐AgNPS, and GCY‐AgNPS) with binding affinities of −13.20, −14.66, and −15.00 kcal/mol, respectively (Tables 4, 5, and 6) looked into molecular docking receptors and the inhibition of *α*‐amylase, as a therapeutic modification approach of diabetes management, by supervising the postprandial blood glucose levels through carbohydrate metabolism delay. Concerning hydrophilic interactions, AgNPs established 13 hydrogen bonds with residues Gln41, Arg195, Val234, Glu255, Phe295, Asn298, His299, Asp300, Arg337, Met339, Val294, Tyr231, and Leu293 (Figures 6a, 6b, 6c). Y‐AgNPS established 14 hydrogen bonds incorporating His15, Phe17, Gln41, Tyr62, Arg195, Tyr231, Thr254, Glu255, Asn298, Arg337, Asp96, Ile13, and His299, and Glu233 (Figures 7a, 7b, 7c). GCY‐AgNPS has the highest number of interactions, with 17 hydrogen bonds formed with His15, Val42, Gln41, Tyr62, Arg195, Asn298, His299, Asp300, Arg337, Ser340, Glu233, Met339, Asp297, Asp96, Val338, Tyr94, and Tyr231 (Figure 8a, 8b, and 8c). Concerning hydrophobic interactions, AgNPs established six carbon‐hydrogen bonds with (His15, Arg195, Phe256, Leu293, His299, Arg337), one *π*‐donor hydrogen bond (Tyr231), and multiple metal–acceptor interactions (Arg337, Leu293, Val42, Asp96, Gln41). Y‐AgNPS formed eight carbon‐hydrogen bonds (His15, Asp197, Glu233, Asn298, His299, Arg337, Tyr231, Tyr94) in addition to metal‐acceptor interactions (Tyr231, Leu293, Val42, Asp96, Gln41). GCY‐AgNPS demonstrated three carbon‐hydrogen bonds (Ser43, Leu293, His299) and five *π*‐donor hydrogen bonds (His15, Phe295, Phe256), thus contributing to the stability of the complex.

**Table 4:** Molecular interactions of AgNPs with the amino acid residues of the selected targets.

**No**	**Proteins**	**3D structure**	**Hydrophilic interactions**		**Hydrophobic contacts**		**No. of H-bonds**	**No. of total bonds**	**Affinity (kcal mol** ^**-1**^ **)**
			**Residue (H- bond)**	**Length (Å)**	**Residue (bond type)**	**Length (Å)**			
1	Amylase		Gln41 (H‐ Bond)	2.61	His15, (Carbon‐H bond)	3.35	13	22	−13.20
			Arg195 (H‐ Bond)	1.82	Arg195, (Carbon‐H bond)	2.70			
			Val234 (H‐ Bond)	2.68	Phe256, (Carbon‐H bond)	3.01			
			Glu255 (H‐ Bond)	1.78	Leu293, (Carbon ‐H bond)	2.71			
			Phe295 (H‐ Bond)	2.90	His299,(Carbon ‐H bond)	2.32			
			Asn298 (H‐ Bond)	2.15	Arg337, (Carbon ‐H bond)	2.74			
			His299 (H‐ Bond)	1.75	Arg337,(Metal‐ Acceptor)	2.81			
			Asp300 (H‐ Bond)	2.20	His15,(Pi‐Donor H Bond)	3.77			
			Arg337 (H‐ Bond)	2.13	Tyr231,(Pi‐Donor H Bond)	2.74			
			Met339 (H‐ Bond)	3.33					
			Val294 (H‐ Bond)	2.62					
			Tyr231 (H‐ Bond)	2.50					
			Leu293 (H‐ Bond)	2.76					

2	*DPP4*		Ser209 (H‐ Bond)	3.33	Tyr585, (Metal‐Acceptor)	3.32	6	10	−9.00
			Arg125 (H‐ Bond)	3.38	Tyr547, (Metal‐Acceptor)	3.10			
			Gln553 (H‐ Bond)	3.16	Phe357, Pi‐Donor H Bond)	3.39			
			Tyr547 (H‐ Bond)	3.13	Phe357, Pi‐Donor H Bond)	3.48			
			Tyr585 (H‐ Bond)	2.73					
			Cys551 (H‐ Bond)	3.10					

3	*GLP-1R*		Tyr42 (H‐ Bond)	2.02	Arg64, (Carbon‐H bond)	2.87	10	18	−15.80
			Arg48 (H‐ Bond)	2.96	Ala70, (Carbon‐H bond)	2.94			
			Cys71 (H‐ Bond)	1.91	Trp72, (Carbon‐H bond)	1.61			
			Ser84 (H‐ Bond)	2.87	Pro73, (Carbon‐H bond)	2.34			
			Asp73 (H‐ Bond)	3.36	Gln45, (Metal‐Acceptor)	2.87			
			Gln45 (H‐ Bond)	2.45	Cys71, (Metal‐Acceptor)	2.37			
			Ser49 (H‐ Bond)	2.28	Ser49, (Metal‐Acceptor)	3.23			
			Asp53 (H‐ Bond)	2.37	Cys46, (Metal‐Acceptor)	2.45			
			Asn82 (H‐ Bond)	2.74					
			Cys46 (H‐ Bond)	2.74					

4	*PPARγ*		Leu228, (H‐Bond)	1.98	Phe226, (Carbon‐H bond)	3.20	13	21	−10.60
			Arg288, (H‐Bond)	2.11	Pro227, (Carbon‐H bond)	2.40			
			Glu291, (H‐Bond)	2.22	Ser289, (Carbon‐H bond)	3.42			
			Ala292, (H‐Bond)	2.67	Leu330, (Carbon‐H bond)	3.04			
			Val293, (H‐Bond)	2.58	Glu295, (Metal‐ Acceptor)	3.16			
			Leu333, (H‐Bond)	2.32	Glu343,(Metal‐Acceptor)	2.73			
			Ser342, (H‐Bond)	2.38	Met329,(Metal‐Acceptor)	3.26			
			Glu343, (H‐Bond)	2.85	Phe387,(Pi‐Donor H Bond)	3.58			
			Leu228, (H‐Bond)	3.31					
			Glu343, (H‐Bond)	3.13					
			Leu340, (H‐Bond)	3.16					
			Glu295, (H‐Bond)	2.22					
			Ser289, (H‐Bond)	3.29					

5	*SGLT2*		Ser287, (H‐Bond)	3.35	Gly83, (Carbon‐H bond)	3.74	4	8	−10.00
			Gln457, (H‐Bond)	2.49	His80, (Carbon‐H bond)	2.79			
			Ser460, (H‐Bond)	2.73	Ser460, (Carbon‐H bond)	3.09			
			Gly79, (H‐Bond)	2.69	Phe98, (Pi‐Donor H Bond)	3.63			

Abbreviations: Ala: alanine, Arg: arginine, Asn: asparagine, Asp: aspartic acid, Cys: cysteine, Glu: glutamic acid, Gln: glutamine, Gly: glycine, His: histidine, Ile: isoleucine, Leu: leucine, Lys: lysine, Met: methionine, Phe: phenylalanine, Pro: proline, Ser: serine, Thr: threonine, Trp: tryptophan, Tyr: tyrosine, Val: valine.

**Table 5:** Molecular interactions of Y‐AgNPS with the amino acid residues of the selected targets.

**No**	**Proteins**	**3D structure**	**Hydrophilic interactions**		**Hydrophobic contacts**		**No. of H-bonds**	**No. of total bonds**	**Affinity (kcal mol** ^**-1**^ **)**
			**Residue (H- bond)**	**Length (Å)**	**Residue (bond type)**	**Length (Å)**			
1	Amylase		His15 (H‐ Bond)	2.63	His15, (Carbon‐H bond)	3.03	14	27	−14.66
			Phe17 (H‐ Bond)	3.29	Asp197, (Carbon‐H bond)	3.36			
			Gln41 (H‐ Bond)	2.88	Glu233, (Carbon‐H bond)	2.85			
			Tyr62 (H‐ Bond)	2.88	Asn298, (Carbon ‐H bond)	2.77			
			Arg195 (H‐ Bond)	2.28	His299,(Carbon ‐H bond)	3.21			
			Tyr231 (H‐ Bond)	2.79	Arg337, (Carbon ‐H bond)	2.88			
			Thr254 (H‐ Bond)	3.24	Tyr231, (Carbon ‐H bond)	2.13			
			Glu255 (H‐ Bond)	2.54	Tyr94, (Carbon ‐H bond)	3.11			
			Asn298 (H‐ Bond)	2.89	Tyr231,(Metal‐Acceptor)	3.37			
			Arg337 (H‐ Bond)	2.91	Leu293,(Metal‐Acceptor)	2.95			
			Asp96 (H‐ Bond)	2.98	Val42,(Metal‐Acceptor)	3.30			
			Ile13 (H‐ Bond)	3.05	Asp96,(Metal‐Acceptor)	3.38			
			His299 (H‐ Bond)	2.73	Gln41,(Metal‐Acceptor)	4.04			
			Glu233 (H‐ Bond)	2.34					

2	*DPP4*		Ser209 (H‐ Bond)	2.98	Ser209, (C‐H‐Bond)	3.29	8	12	−9.11
			Arg358 (H‐ Bond)	2.54	Ser458, (C‐H‐Bond)	3.79			
			Arg429 (H‐ Bond)	3.19	Tyr585, Pi‐Donor H Bond)	4.01			
			Tyr456 (H‐ Bond)	2.67	Phe357, Pi‐Donor H Bond)	3.75			
			Ser458 (H‐ Bond)	3.31					
			Arg471 (H‐ Bond)	3.22					
			Tyr585 (H‐ Bond)	3.20					
			Glu408 (H‐ Bond)	3.36					

3	*GLP-1R*		Tyr42 (H‐ Bond)	2.80	Tyr42, (Carbon‐H bond)	2.60	15	33	−16.88
			Thr65 (H‐ Bond)	2.20	Arg43, (Carbon‐H bond)	2.80			
			Asp67 (H‐ Bond)	2.64	Cys46, (Carbon‐H bond)	2.22			
			Glu68 (H‐ Bond)	3.07	Ser49, (Carbon‐H bond)	2.75			
			Cys71 (H‐ Bond)	2.46	Leu50, (Carbon ‐H bond)	2.36			
			Trp72 (H‐ Bond)	2.55	Arg64,(Carbon ‐H bond)	2.36			
			Val83 (H‐ Bond)	2.55	Phe66, (Carbon ‐H bond)	3.05			
			Ser84 (H‐ Bond)	2.37	Ala70, (Carbon ‐H bond)	2.77			
			Trp87 (H‐ Bond)	2.99	Cys71, (Carbon ‐H bond)	2.56			
			Val100 (H‐ Bond)	2.71	Trp72, (Carbon ‐H bond)	3.06			
			Cys46 (H‐ Bond)	2.61	Pro73,(Carbon ‐H bond)	3.09			
			Asn82 (H‐ Bond)	3.19	Trp72, (Metal‐Acceptor)	2.89			
			Asp53 (H‐ Bond)	2.38	Tyr42,(Metal‐Acceptor)	2.19			
			Ser49 (H‐ Bond)	3.06	Thr65,(Metal‐Acceptor)	2.05			
			Cys46 (H‐ Bond)	3.09	Pro73,(Metal‐Acceptor)	2.42			
					Thr65,(Metal‐Acceptor)	3.03			
					Ala70,(Metal‐Acceptor)	2.58			
					Cys71,(Metal‐Acceptor)	2.71			

4	*PPARγ*		Leu228, (H‐Bond)	1.98	Cys285, (Carbon‐H bond)	2.38	13	26	−11.90
			Ser289, (H‐Bond)	2.11	Arg288, (Carbon‐H bond)	2.61			
			Ala292, (H‐Bond)	2.22	Ser289, (Carbon‐H bond)	2.70			
			Met329, (H‐Bond)	2.67	Ile326, (Carbon‐H bond)	2.69			
			Leu330, (H‐Bond)	2.58	Tyr326, (Carbon ‐H bond)	2.62			
			Leu333, (H‐Bond)	2.32	Tyr327,(Carbon ‐H bond)	2.97			
			Ser342, (H‐Bond)	2.38	Leu330, (Carbon ‐H bond)	1.92			
			Glu343, (H‐Bond)	2.85	Ile341, (Carbon ‐H bond)	3.27			
			Leu340, (H‐Bond)	3.31	Cys285, (Metal‐Acceptor)	3.21			
			Ile326, (H‐Bond)	3.13	Met329,(Metal‐Acceptor)	2.63			
			Ile325, (H‐Bond)	3.16	Glu295,(Metal‐Acceptor)	3.30			
			Glu295, (H‐Bond)	2.22	Arg288,(Metal‐Acceptor)				
			Leu285, (H‐Bond)	3.29	Met364,(Metal‐Acceptor)				

5	*SGLT2*		Asn75, (H‐Bond)	3.13	Gly79, (Carbon‐H bond)	3.68	9	18	−10.55
			Asp158, (H‐Bond)	2.90	His80, (Carbon‐H bond)	3.09			
			Gln457, (H‐Bond)	3.31	Ile76, (Carbon‐H bond)	3.68			
			Glu99, (H‐Bond)	2.70	Lys154, (Carbon ‐H bond)	3.41			
			Gly79, (H‐Bond)	2.94	TYR290,(Carbon ‐H bond)	3.38			
			His80, (H‐Bond)	2.99	Asn75, (Metal‐Acceptor)	2.83			
			Lys154, (H‐Bond)	3.39	Asp158,(Metal‐Acceptor)	3.16			
			Phe98, (H‐Bond)	3.32	Lys154,(Metal‐Acceptor)	2.54			
			Thr153, (H‐Bond)	3.19	Thr153,(Metal‐Acceptor)	2.84			

Abbreviations: Ala: alanine, Arg: arginine, Asn: asparagine, Asp: aspartic acid, Cys: cysteine, Glu: glutamic acid, Gln: glutamine, Gly: glycine, His: histidine, Ile: isoleucine, Leu: leucine, Lys: lysine, Met: methionine, Phe: phenylalanine, Pro: proline, Ser: serine, Thr: threonine, Trp: tryptophan, Tyr: tyrosine, Val: valine.

**Table 6:** Molecular interactions of GCY‐AgNPS with the amino acid residues of the selected targets.

**No**	**Proteins**	**3D structure**	**Hydrophilic interactions**		**Hydrophobic contacts**		**No. of H-bonds**	**No. of total bonds**	**Affinity (kcal mol** ^**-1**^ **)**
			**Residue (H- bond)**	**Length (Å)**	**Residue (bond type)**	**Length (Å)**			
1	Amylase		His15 (H‐ Bond)	2.11	Ser43, (Carbon‐H bond)	3.59	17	25	−15.00
			Val42 (H‐ Bond)	2.68	Leu293, (Carbon‐H bond)	2.99			
			Gln41 (H‐ Bond)	2.93	His299, (Carbon‐H bond)	2.69			
			Tyr62 (H‐ Bond)	2.03	His15,(Pi‐Donor H Bond)	3.00			
			Arg195 (H‐ Bond)	2.89	Phe295,(Pi‐Donor H Bond)	3.82			
			Asn298 (H‐ Bond)	2.44	Phe256,(Pi‐Donor H Bond)	3.35			
			His299 (H‐ Bond)	2.47	Phe295,(Pi‐Donor H Bond)	2.95			
			Asp300 (H‐ Bond)	2.13	His15,(Pi‐Donor H Bond)	2.33			
			Arg337 (H‐ Bond)	2.08					
			Ser340 (H‐ Bond)	2.49					
			Glu233 (H‐ Bond)	3.05					
			Met339 (H‐ Bond)	2.71					
			Asp297 (H‐ Bond)	3.11					
			Asp96 (H‐ Bond)	3.34					
			Val338 (H‐ Bond)	2.55					
			Tyr94 (H‐ Bond)	2.72					
			Tyr231 (H‐ Bond)	2.79					

2	*DPP4*		Arg429 (H‐ Bond)	3.32	Ser552, (C‐H‐Bond)	3.52	8	12	−11.60
			Tyr456 (H‐ Bond)	3.26	Cys551, (Metal‐Acceptor)	3.09			
			Tyr456 (H‐ Bond)	2.87	Tyr585, (Metal‐Acceptor)	3.37			
			Tyr456 (H‐ Bond)	3.36	Gln553, (Metal‐Acceptor)	3.34			
			Tyr547 (H‐ Bond)	2.78					
			Arg560 (H‐ Bond)	2.93					
			Tyr585 (H‐ Bond)	2.69					
			Tyr585 (H‐ Bond)	2.34					

3	*GLP-1R*		Tyr42 (H‐ Bond)	2.68	Tyr42, (Carbon‐H bond)	2.98	17	38	−20.88
			Tyr42 (H‐ Bond)	3.01	Arg43, (Carbon‐H bond)	2.90			
			Thr65 (H‐ Bond)	2.95	Cys46, (Carbon‐H bond)	2.80			
			Ser84 (H‐ Bond)	2.10	Cys46, (Carbon‐H bond)	2.56			
			Ser84 (H‐ Bond)	2.64	Gln47, (Carbon ‐H bond)	2.95			
			Cys71 (H‐ Bond)	1.74	Ser49,(Carbon ‐H bond)	2.61			
			Cys71 (H‐ Bond)	2.15	Ser49, (Carbon ‐H bond)	2.87			
			Trp87 (H‐ Bond)	2.68	Leu50, (Carbon ‐H bond)	2.65			
			Thr65 (H‐ Bond)	3.05	Arg64, (Carbon ‐H bond)	2.16			
			Ser84 (H‐ Bond)	3.35	Trp72, (Carbon ‐H bond)	2.87			
			Trp87 (H‐ Bond)	3.06	Pro73,(Carbon ‐H bond)	3.01			
			Asp53 (H‐ Bond)	3.16	Ser84,(Carbon ‐H bond)	2.71			
			Tyr42 (H‐ Bond)	3.14	Trp87,(Carbon ‐H bond)	1.96			
			Gln45 (H‐ Bond)	2.91	Cys46, (Metal‐Acceptor)	2.86			
			Asp53 (H‐ Bond)	2.66	Cys46,(Metal‐Acceptor)	2.71			
			Gln45 (H‐ Bond)	2.23	Ser49,(Metal‐Acceptor)	2.73			
			Cys71 (H‐ Bond)	3.01	Cys71,(Metal‐Acceptor)	2.66			
					Cys71,(Metal‐Acceptor)	1.28			
					Tyr42,(Pi‐Donor H Bond)	3.14			
					Tyr42,(Pi‐Donor H Bond)	2.93			
					Tyr72,(Pi‐Sigma)	3.99			

4	*PPARγ*		Leu228, (H‐Bond)	2.75	Phe226, (Carbon‐H bond)	2.37	15	31	−15.90
			Arg288, (H‐Bond)	2.81	Pro227, (Carbon‐H bond)	2.78			
			Arg288, (H‐Bond)	2.55	Arg288, (Carbon‐H bond)	2.74			
			Arg288, (H‐Bond)	2.12	Ser289, (Carbon‐H bond)	1.67			
			Ala292, (H‐Bond)	2.12	Ala292, (Carbon ‐H bond)	1.90			
			Val293, (H‐Bond)	1.91	Ala292,(Carbon ‐H bond)	2.17			
			Leu333, (H‐Bond)	2.69	Leu330,(Carbon ‐H bond)	2.22			
			Leu333, (H‐Bond)	2.68	Leu330,(Carbon ‐H bond)	2.81			
			Glu343, (H‐Bond)	2.69	Glu343,(Carbon ‐H bond)	2.32			
			Glu343, (H‐Bond)	2.61	Arg288, (Metal‐Acceptor)	2.45			
			Glu295, (H‐Bond)	2.72	Glu291,(Metal‐Acceptor)	2.28			
			Glu295, (H‐Bond)	3.13	Glu295,(Metal‐Acceptor)	2.76			
			Ser225, (H‐Bond)	3.36	Glu343,(Metal‐Acceptor)	1.50			
			Met329, (H‐Bond)	2.65	Glu295,(Metal‐Acceptor)	3.02			
			Leu330, (H‐Bond)	3.19	Arg288, (Metal‐Acceptor)	3.10			
					Phe287,(Pi‐Donor H Bond)	4.16			

5	*SGLT2*		Asn75, (H‐Bond)	2.38	His80, (Carbon‐H bond)	3.78	12	20	−13.55
			His80, (H‐Bond)	2.85	Ser460, (Carbon‐H bond)	3.08			
			Tyr290, (H‐Bond)	3.14	Gln457, (Metal‐Acceptor)	1.30			
			Lys321, (H‐Bond)	3.20	Glu99,(Metal‐Acceptor)	244			
			Gln457, (H‐Bond)	2.82	Tyr290,(Metal‐Acceptor)	3.14			
			Ser460, (H‐Bond)	2.31	His80,(Pi‐Donor H Bond)	4.18			
			Tyr526, (H‐Bond)	3.34	Tyr290,(Pi‐Donor H Bond)	3.78			
			Phe453, (H‐Bond)	2.24	Phe98,(Pi‐Sigma)	3.57			
			Glu99, (H‐Bond)	3.25					
			Thr153, (H‐Bond)	2.23					
			Asn75, (H‐Bond)	3.12					
			Glu99, (H‐Bond)	2.50					

Abbreviations: Ala: alanine, Arg: arginine, Asn: asparagine, Asp: aspartic acid, Cys: cysteine, Glu: glutamic acid, Gln: glutamine, Gly: glycine, His: histidine, Ile: isoleucine, Leu: leucine, Lys: lysine, Met: methionine, Phe: phenylalanine, Pro: proline, Ser: serine, Thr: threonine, Trp: tryptophan, Tyr: tyrosine, Val: valine.

In the second instance, the activity of dipeptidyl peptidase‐4 (DPP‐4), an enzyme that breaks down incretin hormones, which are important for glucose homeostasis, was assessed. Silver NPs exhibited binding affinities of −9.00, −9.11, and −11.60 kcal/mol for AgNPs, Y‐AgNPS, and GCY‐AgNPS, respectively. AgNPs formed six hydrogen bonds, Tyr547, Tyr585, and Cys551 (Figures 6d, 6e, 6f). Y‐AgNPS formed eight hydrogen bonds: Ser209, Arg358, Arg429, Tyr456, and Ser458, Arg471, and Glu408 (Figures 7d, 7e, 7f). GCY‐AgNPS formed eight hydrogen bonds: Arg429, Tyr456, Tyr547, Arg560, and Tyr585 (Figures Figure 8d, Figure 8e, Figure 8f). AgNPs with hydrophobic interactions included two metal‐acceptor bonds with Tyr585 and Tyr547 and two *π*‐donor H bonds with Phe357. Y‐AgNPS included two carbon‐hydrogen bonds from Ser209 and Ser458, one carbon‐hydrogen bond from Phe357, and two *π*‐donor H bonds with Tyr585. GCY‐AgNPs with one carbon‐hydrogen bond from Ser552, and three metal‐acceptor bonds, Cys551, Tyr585, and Gln553.

Thirdly, the study focused on GLP‐1R (Glucagon‐Like Peptide‐1 Receptor), which remains one of the key therapeutic targets for the augmentation of insulin secretion. AgNPs, Y‐AgNPS, and GCY‐AGNPS exhibited binding affinities of −15.80, −16.88, and −20.88 kcal/mol, respectively. AgNPs formed 10 hydrogen bonds with residues Tyr42, Arg48, Cys71, Ser84, Asp73, Gln45, Ser49, Asp53, Asn82, and Cys46 (Figures 6g, 6h, 6i) and other hydrophobic interactions of four carbon‐hydrogen bonds and four metal‐acceptor bonds. Y‐AgNPS formed 15 hydrogen bonds, which include Tyr42, Thr65, Asp67, Glu68, Cys71, Trp72, Val83, Ser84, Trp87, Val100, Cys46, Asn82, Asp53, Ser49, Cys46 (Figures 7g, 7h, 7i), along with hydrophobic interactions to 11 carbon‐hydrogen bonds and six metal‐acceptor bonds. GCY‐AgNPS showed the strongest interaction, forming 17 hydrogen bonds (Figures Figure 8g, Figure 8h, Figure 8i) and extensive hydrophobic interactions with 13 carbon‐hydrogens, five metal‐acceptors, one *π*‐sigma bond, and two *π*‐donor methyl bonds.

Fourthly, docking analysis revealed that PPAR*γ* (a nuclear receptor responsible for glucose and lipid metabolism) had a binding affinity of −10.60, −11.90, and −15.90 kcal/mol for AgNPs, Y‐AgNPS, and GCY‐AgNPS, respectively. AgNPs formed 13 hydrogen bonds (Leu228, Arg288, Glu291, etc.; Figures 6j, 6k, 6l) and multiple hydrophobic interactions. Y‐AgNPS also formed 13 hydrogen bonds as well as extensive carbon‐hydrogen and metal‐acceptor bonds (Figures 7j, 7k, 7l). GCY‐AgNPS formed 15 hydrogen bonds and the highest hydrophobic profile of all three types (Figures Figure 8j, Figure 8k, Figure 8l).

Finally, the interaction with SGLT2, a glucose‐reabsorbing transporter in the kidney, resulted in affinities of −10.00, −10.55, and −13.55 kcal/mol. Hydrogen bonds and stabilizing hydrophobic interactions were formed by AgNPs at four sites: Ser287, Gln457, Ser460, and Gly79 (Figures 6m, 6n, 6o). Y‐AgNPS also participated in multiple carbon‐hydrogen and metal‐acceptor interactions; however, these bonds were enhanced by nine hydrogen bonds (Figures 7m, 7n, 7o). GCY‐AgNPS exhibited the greatest binding, reaching 11 bonds with a diverse array of stabilizing hydrophobic and *π*‐bonds, as well as metal‐acceptor interactions (Figures Figure 8m, Figure 8n, Figure 8o).

To validate the molecular docking, we redocked the native small‐molecule ligands from the crystal structures using Autodock Vina and calculated the binding affinities and RMSD values relative to their experimental poses (Supplementary Table S1). For alpha‐amylase (PDB: 5U3A), the native inhibitor S7J redocked with a binding affinity of −9.50 kcal/mol and an RMSD of 1.20 Å, demonstrating strong agreement with the crystal structure and reflecting its potent inhibitory activity against carbohydrate hydrolysis. For DPP4 (PDB: 4A5S), the native inhibitor N7F redocked with −9.2 kcal/mol and an RMSD of 1.41 Å, consistent with its experimental IC50 of 17 nM as a potent inhibitor targeting incretin degradation. For GLP‐1R (PDB: 4ZGM), the native modulator 97 V redocked with −8.66 kcal/mol and RMSD of 1.23 Å, aligning well with its role as a negative allosteric modulator and validating the protocol for receptor–ligand interactions in this GPCR family. For PPAR*γ* (PDB: 9F7W), bisphenol A (BPA) redocked with −5.8 kcal/mol and a low RMSD of 0.90 Å, consistent with its known weak binding and endocrine‐disrupting effects via nuclear receptor activation. For SGLT2 (PDB: 8HEZ), dapagliflozin redocked with −10.5 kcal/mol and RMSD of 1.65 Å, correlating with its high potency as a glucose reabsorption inhibitor. Overall, these positive controls confirm the docking method’s accuracy, with low RMSDs indicating minimal deviation from experimental poses and affinities correlating with known inhibitory strengths across diverse antidiabetic targets. This strengthens the interpretation of our NP docking results, where GCY‐AgNPs exhibited the strongest predicted affinities across targets, suggesting potential multi‐target antidiabetic efficacy.

## 4. Discussion

Type 2 diabetes mellitus (T2DM) is rapidly emerging as a global health crisis, resulting in severe complications and lowering the quality as well as quantity of life [24]. Despite the various suggested treatments for T2DM, there is still a lack of effective drugs that prevent further decline in *β*‐cell function over time. Since ancient times, medicinal plants have been used in traditional medicine for the prevention and treatment of various diseases, while also contributing to the enhancement of overall health and well‐being [25].

In recent years, there has been growing interest in nanomaterials because of their potential to pave a new path in medicine by enabling highly targeted therapies and controlled drug release. In addition, these materials benefit from advantageous physicochemical characteristics, which enable them to be used in new areas such as cancer treatment and drug delivery, thereby increasing the therapeutic effect and reducing adverse outcomes. In addition, researchers have realized the potential of nanomaterials. This has exponentially increased interest in synthesizing NPs. However, conventional manufacturing methods tend to be quite detrimental to the environment since they release vast amounts of hazardous materials, increasing the likelihood of human health complications, prompting a shift towards a safe, ecological, biogenic approach for NP production [26].

The field of nanomedicine has shown a growing interest in applying biocompatible metal‐based NPs in diabetes. Various studies have reported different types of NPs that can be utilized for treatment purposes [27]. Nevertheless, the antidiabetic effect of pine needle leaf extract‐loaded NPs has not been examined in vivo. Thus, this work seeks to further this research gap by determining the therapeutic effect of intraperitoneal administration of greenly synthesized NPs formulated from pine needle leaf extract in STZ‐induced diabetic rats. Additionally, it focuses on examining the safety of greenly synthesized NPs by assessing their possible toxicological effects.

STZ commonly induces hyperglycemia in Sprague–Dawley rats, leading to significant weight loss attributable to muscle breakdown and protein catabolism in tissues. This weight loss is often accompanied by increased food and water intake, resulting from the destruction of beta cells and subsequent insulin deficiency [28]. Additionally, diabetic rats may lose weight due to the breakdown of structural proteins essential for maintaining normal body mass [29]. The improvements seen in body weight and the conditions of the organs in the groups treated with NPs can be related to the combined influence of the nanoscale size and the bioactive composition [30]. Small sizes facilitate better glucose utilization and metabolic balance by increasing the rate of cellular uptake and tissue penetration, thereby aiding in the utilization of glucose [31]. Furthermore, the phytochemicals encapsulated in the NPs, largely consisting of polyphenols, flavonoids, and terpenes from the pine‐needle extract, possess strong antioxidant and anti‐inflammatory properties that reduce oxidative stress and reduce proteolysis and lipolysis. In turn, all these contribute to the preservation of muscle mass and the organs of diabetic rats [32]. A positive metabolic efficiency could also result from the insulin that is more readily available to the body and the lower gluconeogenesis in the liver, which stabilizes glucose and decreases glucose‐related tissue‐wasting [33]. Some studies suggest that silver NPs (AgNPs) could influence body weight by interfering with nutrient absorption or altering metabolic pathways [34]. Furthermore, other studies have reported a significant reduction in organ sizes, probably due to NP accumulation, which induces oxidative stress and inflammation, impairing liver and kidney functions. These findings align with earlier research on the impact of AgNPs on organ health in male rats [34]. A decrease in kidney weights has also been observed among diabetic control animals, which is associated with the progression of glomerular pathology and renal hypertrophy [35]. During fasting periods, the amounts observed in untreated or treated diabetics were significantly higher than those recorded among non‐diabetic controls. Consequently, STZ destroys pancreatic *β*‐cells, leading to reduced production of endogenous insulin, decreased glucose absorption by tissue cells, and increased toxic effects on blood sugar levels due to selective *β*‐cell destruction and insufficient insulin release [36].

The liver plays a critical role in blood sugar regulation through various mechanisms, such as glycogenesis, glycogenolysis, and gluconeogenesis [37]. Therefore, liver function tests are critical during diabetes management to determine the therapeutic approaches. Results showed that T2DM causes both hepatic dysfunction and renal failure, accompanied by dyslipidemia. These results also indicated that the increase in ALT and AST serum levels is mainly due to the leakage of these enzymes from damaged hepatocytes into the bloodstream [38]. Likewise, Hassan et al. [39] noted an increase in liver enzymes in STZ‐induced diabetic rats. However, after being administered plant‐extract‐synthesized AgNPs, liver enzymes showed remarkable improvement, thus confirming their hepatoprotective effect [39]. At the same time, inflammation within the liver associated with the pathogenesis of diabetes mellitus could be responsible for the decreased albumin levels observed concurrently [40]. An increase in gluconeogenesis, which comes about due to a lack of insulin, has been frequently linked with elevated ALT and AST levels in diabetics [41].

Additionally, abnormal levels of urea, creatinine, and uric acid in the blood are key indicators of renal dysfunction [41]. Specifically, an increased serum urea level may indicate poor renal excretion [42]. Our results revealed higher serum urea and creatinine levels in diabetic STZ rats. However, these levels were decreased among groups treated with NPs, suggesting that renal dysfunction and possible kidney injury in STZ diabetic rats were caused by severe hyperglycemia [41]. This is consistent with Al Khuzaee et al. [43], who showed that plant‐extract‐synthesized silver NPs reduced serum urea and creatinine levels, thus recovering renal function in diabetic rats. However, some studies using chemically synthesized NPs reported nephrotoxicity, which underscores the benefits of using green‐synthesized nanomaterials [43]. In addition to the increase in serum urea and creatinine, histopathological changes indicative of early diabetic nephropathy, such as mesangial expansion and increased mesangial cellularity [41, 44]. These findings align with previous studies that revealed the effectiveness of silver NPs (AgNPs) in lowering blood levels of blood urea, albumin, and creatinine [45].

In diabetes, managing total cholesterol, TGs, and lipoproteins is crucial, as lipid metabolism disruptions are closely associated with cardiovascular complications [46]. Dyslipidemia can result from insulin deficiency or resistance, as insulin inhibits HMG‐CoA reductase, a key enzyme that regulates cholesterol‐rich LDL particle metabolism [47, 48]. In the STZ‐induced diabetic rats, a significant rise was observed in all lipid parameters compared with non‐diabetic rats. These findings align with those reported by Divya and Anand, who similarly observed elevated blood TG levels and abnormal lipoprotein patterns in the diabetic rat model [49]. Additionally, insulin resistance may promote the entry of free fatty acids into hepatocytes, thereby promoting adipose tissue lipolysis [50]. In diabetic rats treated with silver NPs (AgNP). The cholesterol, LDL, and TG levels were significantly decreased; however, HDL levels increased [45]. Siddiqa et al. [51] noted similar lipid‐lowering effects with biogenic AgNPs. They found that synthesized lipid metabolism modulating AgNPs improved antioxidant balance, which is in concordance with our findings [51]. These improvements in the lipid profile parameters could be attributed to impaired fatty acid synthesis, inhibition of tissue lipases by acetyl‐CoA carboxylase, or suppression of TG precursor synthesis. However, despite their improvements, VLDL‐C levels remained markedly higher than those in non‐diabetic rats [38].

Under treatment with Y‐AgNPS and GCY‐AgNPS NPs, healthy rats demonstrated minor increases in TGs and HDL, and they were thought to be adaptive and not pathological in nature. The increase in HDL levels is advantageous as it stimulates reverse cholesterol transport and helps in the antioxidant defense, while the mild increase in TGs is probably due to enhanced lipids being mobilized and mitochondrial turnover [52]. The absence of toxicity is proven by the enzymes of the liver. Hence, healthy rats show that Y‐AgNPS and GCY‐AgNPS NPs dynamically modulate the lipid metabolism, whereas diabetic rats show that Y‐AgNPS and GCY‐AgNPS NPs offer corrective therapeutic effects by dyslipidemia and metabolic balance restoration.

The histopathological findings highlight the nephroprotective potential of green‐synthesized NPs against STZ‐induced renal damage, closely correlating with previous studies, while also revealing new insights related to biochemical markers [53]. The STZ group revealed diabetic nephropathy, including glomerular swelling, loss of PCT, particularly the PCT that limits diffusion and nutrient uptake, and marked cellular inflammation. These changes reflect the classic effects of STZ‐induced oxidative stress and apoptosis on renal tissues. Previous studies have reported similar findings, highlighting that the damage originates from inflammation in the renal cortex and subsequent degeneration of glomerular and tubular structures due to STZ‐induced oxidative stress [29, 42–44]. However, cortical and tubular changes were less noticeable in NP‐administered groups; in particular, STZ + Ag, STZ + Y‐AgNPS, and STZ + GCY‐AgNPS exhibited more favorable outcomes, such as an increased glomerular and tubular size and reduced inflammatory infiltration. This aligns with El‐Demerdash et al., where AgNPs helped to restore glomerular architecture and decreased the amount of tubular necrosis present in diabetic kidneys [54]. In addition, this is consistent with the literature, which states that metallic NPs, particularly silver (Ag), possess the ability to scavenge free radicals and exert anti‐inflammatory effects, thereby protecting renal tissues from oxidative damage [55–57]. Furthermore, the biogenic NPs exhibited protective actions against STZ toxicity, preserving renal function in diabetic models, particularly in the GCY‐AgNPS group. These findings accord well with previous studies showing the anti‐inflammatory and antioxidant effects of green‐synthesized NPs, resulting in reduced cellular damage, inflammation, and necrosis [46, 47].

In the present investigation, the protective effects of the nanoparticles are further corroborated by the biochemical markers, which indicated enhancement of the renal function parameters such as serum creatinine, urea, and blood urea nitrogen (BUN) in the NP‐treated groups. The improvement in the kidney structure, including the intact glomeruli and the reduced tubular necrosis, is also consistent with previous studies reporting that restored kidney filtration is associated with less oxidative stress [58]. The association can be explained through the critical roles of glomeruli and the PCT in renal activities [58]. Selectively, the glomeruli allow free circulation of water, electrolytes, urea, and creatinine to pass into the filtrate while preventing proteins from leaking out. A selective filtration process is crucial for maintaining homeostasis and regulating waste elimination [59]. Impairments in the glomerular structure directly reduce filtration efficiency, leading to increased levels of creatinine and urea biochemical markers in the blood, which imply kidney malfunction [60]. Furthermore, the PCT contributes mainly to the reabsorption of filtered nutrients, water, and ions and the secretion of waste products. Additionally, PCT injury, such as tubular necrosis, inhibits reabsorption and secretion processes, worsening biochemical imbalances [22, 41]. There is evidence that oxidative stress plays a significant role in the pathogenesis of kidney disease involving glomerular and tubular function [61, 62]. Nanoparticles with antioxidative properties lessen oxidative stress, preserving kidney structure and function, thereby preventing damage to renal tissues and improving overall kidney health, potentially reducing the risk of kidney damage associated with various renal disorders. Several studies show that nanoparticles lower oxidative stress markers, such as creatinine and urea, and improve kidney filtration function in animal models [34]. The STZ + PNLE group showed moderate protection, with some renal injury remaining. This implies that although plant‐based nanoparticles (PNLE) exhibit antioxidant properties, they may be less effective than metallic NPs in reducing the severity of renal injury. Glibenclamide is a first‐choice treatment for managing hyperglycemia‐induced renal complications via promoting insulin secretion and reducing oxidative stress [63]. Therefore, histopathological and biochemical investigations suggest that greenly synthesized nanoparticles can potentially treat diabetic nephropathy.

The molecular docking results suggest that all variants of silver nanoparticles contain favorable binding interactions with major protein anti‐diabetic targets, with GCY‐AgNPs having the greatest binding affinities and largest interaction networks as compared with the rest. For *α*‐amylase, the gradual rise in hydrogen bonding and hydrophobic interactions over AgNPs, Y‐AgNPs, and GCY‐AgNPs reflects greater adherence, compatibility, and stabilization at the molecular level within the catalytic site. Asn298, His299, Leu293, and Tyr94 were recurring across all the nanoparticle variants, which signifies their importance in binding. This confirms the work of Siddique et al., who found similar important residues during their docking simulations with nanoparticles [64].

For DPP‐4, the commonly known residues Ser209, Ser458, and Tyr585 were important in preserving the stability of ligands within all complexes. We stated that the relatively lower binding energies in comparison with *α*‐amylase could result from the differences in pocket size and the electrostatic environment. Regardless, the data corroborate Thabet et al., reinforcing the potential of inhibition at multiple glycemic targets with nanoparticles [65]. In addition, GLP‐1R interactions discovered that the count of hydrogen bonds had a strong dependence on the binding affinity. Notably, GCY‐AgNPS outperformed both AgNPs and Y‐AgNPS, suggesting the functionalization had a synergistic effect on the receptor interaction. Some of the shared residues, Cys46, Cys71, and Phe66, highlight preserved binding hot spots, which also argues for Sharma et al. regarding structural nanointeractions in GLP‐1R modulation [66].

Concerning PPAR*γ*, the predominance of GCY‐AgNPS in both binding affinity and interaction complexity (for instance, Arg288, Glu343, Leu330) aligns with Wong et al., who actively highlighted the preference of functionalized nanomaterials in regulating nuclear receptors [67]. The detection of recurring interaction residues, like Ser289 and Leu333, continues to support the therapeutic hopes of targeting PPAR*γ* through nanoparticle‐based platforms.

SGLT2 docking results confirm the ability of AgNPs to modulate renal glucose handling, with GCY‐AgNPs exhibiting the strongest interactions, especially at His80, Glu99, and Tyr290. These residues, consistently recurring across all variants, corroborate the conclusions of Wang et al. regarding the importance of these residues in nanoparticle−SGLT2 interactions as the dominant region of binding [68]. As noted above, the remarkable GCY‐AgNPS outperformance on each molecular target showcased their greater bioactivity, as well as therapeutic potential. These in silico outcomes demand additional testing in vitro and in vivo to assess their potential usefulness in strategies aimed toward anti‐diabetic therapies.

## 5. Conclusion

This study implies that eco‐friendly nanoparticles have significant antihyperglycemic and anti‐hyperlipidemic actions, improving biochemical profiles and reducing diabetic complications. This global move for sustainability, supported by cheapness, should promote the use of common medicinal plants as templates for synthesizing nanoparticles with different pharmacological properties. Therefore, these particles are recommended as effective nanomedicines for treating diabetes mellitus and hyperlipidemia. Additionally, our investigation brings out other uses of green‐synthesized NPs in managing or preventing diabetic nephropathy‐induced complications; however, more research is needed to understand their complete molecular mechanisms at cellular and physiological levels. The efficiency of NPs was further confirmed by computational docking studies, which demonstrated strong binding affinities to pivotal anti‐diabetic targets (*α*‐amylase, DPP‐4, GLP‐1R, PPAR*γ*, SGLT2) via multiple hydrogen, hydrophobic, and *π*‐donor interactions, highlighting their multi‐target action scope of a therapeutic agent and triadic pharmacology. The results sustain NPs as promising multifunctional and environmentally friendly agents for the management of diabetes and integrate the ancient wisdom of medicine with modern nanotechnology. All investigated formulations of nanoparticles showed that GCY‐AgNPS had the best overall efficacy and demonstrated the best therapeutic performance of all tested nanoparticles. The findings demonstrate the bridging of the ancient practice of medicine with modern nanotechnology.

Nonetheless, this study recognizes constraints in terms of a smaller sample size, shorter duration of treatment, and lack of mechanistic validation at the molecular and gene‐expression levels. These first‐of‐the‐kind studies should incorporate larger study populations and potentially broaden to include molecular pathways and subsequent clinical pathways to assure the claimed safety and therapeutic efficacy, in the long term, of the green‐synthesized nanoparticles.

## Disclosure

All authors have read and agreed to the submitted version of the manuscript.

## Conflicts of Interest

The authors declare no conflicts of interest.

## Author Contributions

**Nourhane A. Darwich:** investigation, conceptualization, validation, methodology, data curation, formal analysis, software, writing – review & editing original draft; **Noura S. Abouzeinab:** methodology, data curation, formal analysis, software, writing – review & editing original draft; **Ahmed F. El-Sayed:** methodology, data curation, formal analysis; software, writing – review & editing original draft; **Rana El Hajj:** conceptualization, validation, writing – review & editing, supervision; **Mahmoud I Khalil:** investigation, conceptualization, methodology, formal analysis, validation, writing – review & editing, main supervision. Nourhane A. Darwich and Noura S. Abouzeinab contributed equally to this work.

## Funding

No funding was received for this manuscript.

## Supporting information


**Supporting Information** Additional supporting information can be found online in the Supporting Information section. Table S1: List of targets, PDB IDs, resolution, and active site coordinates. Table S2: Average body weight (g) of experimental groups at different time points (Day 7 and Day 21).

## Data Availability

The data that support the findings of this study are available from the corresponding author upon reasonable request.
