# Supplementary material for: In Vivo Therapeutic Potential of Biologically Synthesized Nanoparticles From Pine Needle Leaf Extract in Streptozotocin‐Induced Diabetic Rats
Source: Biomed Res Int. 2026 Jan 21;2026:1938383. doi: 10.1155/bmri/1938383 (PMC12824417; doi:10.1155/bmri/1938383)
Supplement: Supplementary file 1 — Supporting Information Additional supporting information can be found online in the Supporting Information section. Table S1: List of targets, PDB IDs, resolution, and active site coordinates. Table S2: Average body weight (g) of experimental groups at different time points (Day 7 and Day 21). [file BMRI-2026-1938383-s001.docx]

*In vivo* Therapeutic Potential of Biologically Synthesized Nanoparticles from Pine Needle Leaf Extract in Streptozotocin-Induced Diabetic Rats

**Supplementary information**

**Supplementary Table S1.** List of targets, PDB IDs, resolution, and active site coordinates.

| NO | Protein Targets | PDB ID | Resolution | Active site coordinates: | | | Native Ligand  (Positive control) | Affinity (kcal/mol) | RMSD (Å) | Reference |
| --- | --- | --- | --- | --- | --- | --- | --- | --- | --- | --- |
|  |  |  |  | X | Y | Z |  |  |  |  |
| 1 | Human Pancreatic alpha-amylase | 5U3A | 1.20 Å | 16.36 | 15.09 | 41.96 | **5J7** (5,7-dihydroxy-4-oxo-2-(3,4,5-trihydroxyphenyl)-4H-chromen-3-yl 6-deoxy-2-O-{6-O-[(2E)-3-(3,4-dihydroxyphenyl)prop-2-enoyl]-beta-D-glucopyranosyl}-alpha-L-mannopyranoside) | -9.50 | 1.20 | [72] |
| 2 | DPP4 (Dipeptidyl Peptidase-4) | 4A5S | 1.62 Å | 23.46 | 33.71 | 57.71 | **N7F** (6-[(3S)-3-aminopiperidin-1-yl]-5-benzyl-4-oxo-3-(quinolin-4-ylmethyl)-4,5-dihydro-3H-pyrrolo[3,2-d]pyrimidine-7-carbonitrile) | -9.20 | 1.41 | [73] |
| 3 | GLP-1R (Glucagon-Peptide-1 Receptor) | 4ZGM | 1.80 Å | 13.42 | 50.29 | 3.66 | **97Y** (N-{4-[(R)-(3,3-dimethylcyclobutyl)({6-[4-(trifluoromethyl)-1H-imidazol-1-yl]pyridin-3-yl}amino)methyl]benzene-1-carbonyl}-beta-alanine) | -8.66 | 1.25 | [74] |
| 4 | Peroxisome Proliferator-Activated Gamma | 9F7W | 1.25 Å | 47.80 | 11.18 | 14.82 | **BPA** (Bisphenol A) | -5.80 | 0.90 | [75] |
| 5 | SGLT2 (Sodium-Glucose Transporter-2) | 8HEZ | 2.80 Å | 69.70 | 67.01 | 72.01 | **LE6** (Dapagliflozin) | -10.50 | 1.65 | [76] |

**Supplementary Table S2.** Average body weight (g) of experimental groups at different time points (Day 7 and Day 21).

| **Experimental groups** | **N** | **Time** | | |
| --- | --- | --- | --- | --- |
|  |  | **7^rd^ day** | **21^st^ day** | **Results of one-way ANOVA (Time)** |
| **Control** | 3 | 164.66±9.82^a,*^ | 274.33±24.60^#^ |  |
|  |  |  |  | **F=17.135 p<0.05 (0.014)** |
| **STZ** | 3 | 190.33±5.48^ab^ | 177.33±34.83 |  |
|  |  |  |  | **F=0.136 p>0.05** |
| **STZ+AgNPs** | 3 | 197.33±11.20^bcd^ | 237.00±27.49 |  |
|  |  |  |  | **F=1.785 p>0.05** |
| **STZ+Y-AgNPs** | 3 | 213.00±14.43^bc^ | 256.33±6.17 |  |
|  |  |  |  | **F=7.619 p>0.05** |
| **STZ+GCY-AgNPs** | 3 | 214.00±11.01^bc^ | 252.33±12.77 |  |
|  |  |  |  | **F=5.166 p>0.05** |
| **STZ+PNLE** | 3 | 222.33±7.21^c^ | 238.66±8.66 |  |
|  |  |  |  | **F=2.097 p>0.05** |
| **GLB** | 3 | 213.66±10.58^bc^ | 244.66±17.93 |  |
|  |  |  |  | **F=2.215 p>0.05** |
| **AgNPs** | 3 | 178.66±4.17^ad,*^ | 216.66±8.87^#^ |  |
|  |  |  |  | **F=15.007 p<0.05 (0.018)** |
| **Y-AgNPs** | 3 | 198.00±8.50^bcd^ | 241.66±13.48 |  |
|  |  |  |  | **F=7.504 p>0.05** |
| **GCY-AgNPs** | 3 | 184.00±11.35^ad,*^ | 235.00±8.96^#^ |  |
|  |  |  |  | **F=12.425 p<0.05 (0.024)** |
| **PNLE** | 3 | 174.33±6.76^ad,*^ | 230.33±10.68^#^ |  |
|  |  |  |  | **F=19.614 p<0.05 (0.011)** |
| **Results of one-way ANOVA (group)** | |  |  |  |
|  |  | **F=3.864**  **P<0.05 (0.004)** | **F=1.852**  **P>0.05** |  |
